# *MYB* Elongation Is Regulated by the Nucleic Acid Binding of NFκB p50 to the Intronic Stem-Loop Region

**DOI:** 10.1371/journal.pone.0122919

**Published:** 2015-04-08

**Authors:** Lloyd A. Pereira, Honor J. Hugo, Jordane Malaterre, Xu Huiling, Secondo Sonza, Alina Cures, Damian F. J. Purcell, Paul A. Ramsland, Steven Gerondakis, Thomas J. Gonda, Robert G. Ramsay

**Affiliations:** 1 Differentiation and Transcription Laboratory, Peter MacCallum Cancer Centre, Locked Bag #1, Melbourne, Victoria, 8006, Australia; 2 Sir Peter MacCallum Department of Oncology, The University of Melbourne, Parkville, Victoria, 3010, Australia; 3 The Department of Pathology, The University of Melbourne, Parkville, Victoria, 3010, Australia; 4 Victorian Breast Cancer Consortium, Invasion and Metastasis Unit, St Vincent’s Institute of Medical Research, Melbourne, Victoria, 3065, Australia; 5 The Department of Microbiology and Immunology, The University of Melbourne, Parkville, Victoria, 3010, Australia; 6 Centre for Immunology, Burnet Institute, Melbourne, Victoria, 3004, Australia; 7 Department of Surgery (Austin Health), The University of Melbourne, Heidelberg, Victoria, 3084, Australia; 8 Department of Immunology, Monash University, Alfred Medical Research and Education Precinct, Melbourne, Victoria, 3004, Australia; 9 Australian Centre for Blood Diseases, Monash University, Prahran, Victoria 3004, Australia; 10 School of Pharmacy University of Queensland, Woolloongabba, Queensland, 4102, Australia; CNRS UMR7275, FRANCE

## Abstract

*MYB* transcriptional elongation is regulated by an attenuator sequence within intron 1 that has been proposed to encode a RNA stem loop (SLR) followed by a polyU tract. We report that NFκBp50 can bind the SLR polyU RNA and promote *MYB* transcriptional elongation together with NFκBp65. We identified a conserved lysine-rich motif within the Rel homology domain (RHD) of NFκBp50, mutation of which abrogated the interaction of NFκBp50 with the SLR polyU and impaired NFκBp50 mediated *MYB* elongation. We observed that the TAR RNA-binding region of Tat is homologous to the NFκBp50 RHD lysine-rich motif, a finding consistent with HIV Tat acting as an effector of *MYB* transcriptional elongation in an SLR dependent manner. Furthermore, we identify the DNA binding activity of NFκBp50 as a key component required for the SLR polyU mediated regulation of *MYB*. Collectively these results suggest that the *MYB* SLR polyU provides a platform for proteins to regulate *MYB* and reveals novel nucleic acid binding properties of NFκBp50 required for *MYB* regulation.

## Introduction

The *MYB* proto-oncogene encodes a transcription factor that plays an important role in cellular proliferation and differentiation [1]. While much of the pioneering research on *MYB* focused on hematopoiesis and malignancies [1], its expression has subsequently been shown to be important in the context of epithelial cancer biology, most notably breast and colon cancer [1]. *MYB* is essential for the proliferation of ERα positive breast cancer cells [2] and is required for mammary carcinogenesis in murine models [3]. In colorectal cancer (CRC), *MYB* is frequently over-expressed, a property that correlates with poor prognosis for patients with CRC [4]. *MYB* co-operates with Wnt to generate intestinal cancers in mice [5] and drives the expression of the gastrointestinal stem cell genes *Lgr5*, *olfm4* and *Bmi1* [6].

The regulation of transcription elongation has emerged as an important mechanism used to control eukaryotic gene expression [7–14]. In line with this view, accumulated data has revealed the important role of transcriptional elongation in the control of *MYB* expression [1]. In the murine hematopoietic system, a direct correlation exists between the expression levels of *MYB* RNA and transcription elongation arrest (attenuation) that operates in the first intron of the gene [15–19]. Similarly, in CRC lines the rapid down-regulation of *MYB* expression that occurs during induced differentiation by sodium butyrate, was found to result from transcription elongation arrest within intron 1, ~1.7kb downstream of the transcription initiation site [20, 21]. Furthermore, *MYB* expression in breast cancer cells is regulated by a similar mechanism, whereby attenuation is overcome by estrogen receptor binding [2, 22]. We previously proposed that *MYB* elongation arrest was regulated by RNA secondary structure [1, 20, 21]. We modeled the RNA encoded by the attenuation region and showed that it potentially formed an energetically stable RNA stem loop region (SLR) followed by a 1920-ribonucleotide polyU tract [20, 21]. In the case of CRC a high frequency of mutations occur in the *MYB* SLR that correlate with elevated levels of *MYB* mRNA observed in primary CRCs and derived cell lines [20, 21]. These data suggest that the dysregulation of SLR function may be an important component in the over expression of *MYB* in colorectal cancer [1].

Despite the body of data indicating that the *MYB* SLR is an integral mediator of *MYB* transcriptional elongation, our understanding of how the SLR regulates the elongation process remains poor. The implication of NFκB proteins in the regulation of murine *MYB* transcriptional elongation via intron 1 sequences [23–25] led us to consider a role for NFκB proteins in regulating *MYB* via the *MYB* SLR. Here we demonstrate that NFκB proteins p50 and p65 (RelA) promote elongation through the *MYB* intron 1 attenuation region in an SLR and polyU dependent manner. Furthermore, NFκBp50 directly associates with the *MYB* SLR RNA through its amino terminal Rel homology domain (RHD). We identify a conserved lysine rich motif within the RHD, that is homologous to the TAR RNA-binding region of HIV-1 Tat and HEXIM 1 proteins [26, 27]. Mutation of the lysine rich motif abolished p50 interaction with the SLR and impaired NFκBp50-mediated transcriptional elongation through the SLR. Furthermore, we identify the DNA binding activity of NFκBp50 as a key component NFκBp50 mediated stimulation of *MYB* SLR polyU elongation. Collectively these results suggest that the *MYB* SLR and polyU provide a platform for proteins to bind and regulate *MYB* elongation. The nucleic acid binding activity of NFκBp50 that includes its RNA and DNA binding functions may provide a novel mechanism for the regulation of *MYB* expression with important implications for NFκB function in other contexts.

## Materials and Methods

### Cell culture

HEK293 (ATCC CRL-11268) cells were cultured in RPMI1640 10%FBS. The propagation and derivation of LIM1215 cells has been described [6]. Murine colon organoids were generated using previously established protocols [6]. For TNFα stimulation, 293 cells were plated overnight and TNFα (Abbiotec, USA) added at the concentrations specified. For 5,6-Dichloro-1-beta-D-ribofuranosylbenzimidazole (DRB; Sigma) treatment of 293 cells, DRB was dissolved in DMSO as recommended. DRB was diluted in RPMI to a final concentration of 30 μM and added to the cells for the times indicated in the figure legends. For NFκB inhibition, 293 cells were plated overnight and BAY 11–7082 (Millipore) added at the concentrations and times indicated in the figure legends.

### Plasmids

*MYB* CAT reporters have been described [21]. The *MYB* TAR CAT reporter was generated by removing the *SLR* and polyU region from the MYB *SLR* polyCAT reporter via a *Xho*I and *Xba*I digest and subsequently cloning in the HIV-1 TAR sequence. The pBluescriptIIKS-*MYB* SLR polyU, *MYB* ΔSLR polyU, *MYB* SLR ΔpolyU and *MYB* SLR Δ9 polyU were generated by cloning PCR fragments generated from pALTER1-*MYB* into pBluescriptIIKS via *Bam*HI and *Eco*RV sites. pGEM-3Zf *MYB* SLR polyU, *MYB* ΔSLR polyU and *MYB* SLR ΔpolyU were constructed by cloning *MYB* SLR polyU sequences into pGEM via *EcoRI* and *HindIII* sites. pCMVTAT(72) was supplied by Malcolm Martin (NIAID, NIH). pCMV CDK9 and dominant negative pCMV DNCDK9 were gifts from Andy Rice (Baylor College, USA). pcDNA NFκBp50 and NFκBp65 were created by Gateway cloning (Invitrogen) cDNAs into pcDNA3.2/V5 and in the case of NFκBp50 also into pET15b. pCMVβ-galactosidase and pACTCAT*-*β-actin were a gift from Shunsuke Ishii (RIKEN, Japan). pHIVLTRCAT was obtained from the NIH AIDS Research and Reference Reagent Program. WT and bulge mutant *TAR* pGEM-3Zf vectors were a gift of Melanie Ott (Gladstone Institute of Virology and Immunology, USA) [28]. pNFκB p50-65 was a gift of Craig Rosen (Roche Institute of Molecular Biology) [29]. Mutagenesis of NFκBp50 and NFκBp50-65 was performed using the Gene Tailor site-directed mutagenesis system (Invitrogen). All oligonucleotide sequences are provided S1 Table. Ectopic protein expression from all mammalian expression plasmids was confirmed by Western blot analysis S1 Fig.

### CAT reporter assays

293 cells were transfected with 2–3 μg total DNA using Fugene (Roche). pCMVβ-gal was used as a transfection control. Cells were harvested and CAT activity measured as described [6]. Data shown represent an average of at least three experiments.

### Chromatin Immunopreciptation (ChIP) assays

ChIP assays were performed as previously described [6]. Anti-RNA polymerase II (H224X; Santa Cruz) was used to detect RNA polymerase II at the *MYB* SLR polyU.

### Protein Extracts and Western blotting

**Fig 1:**
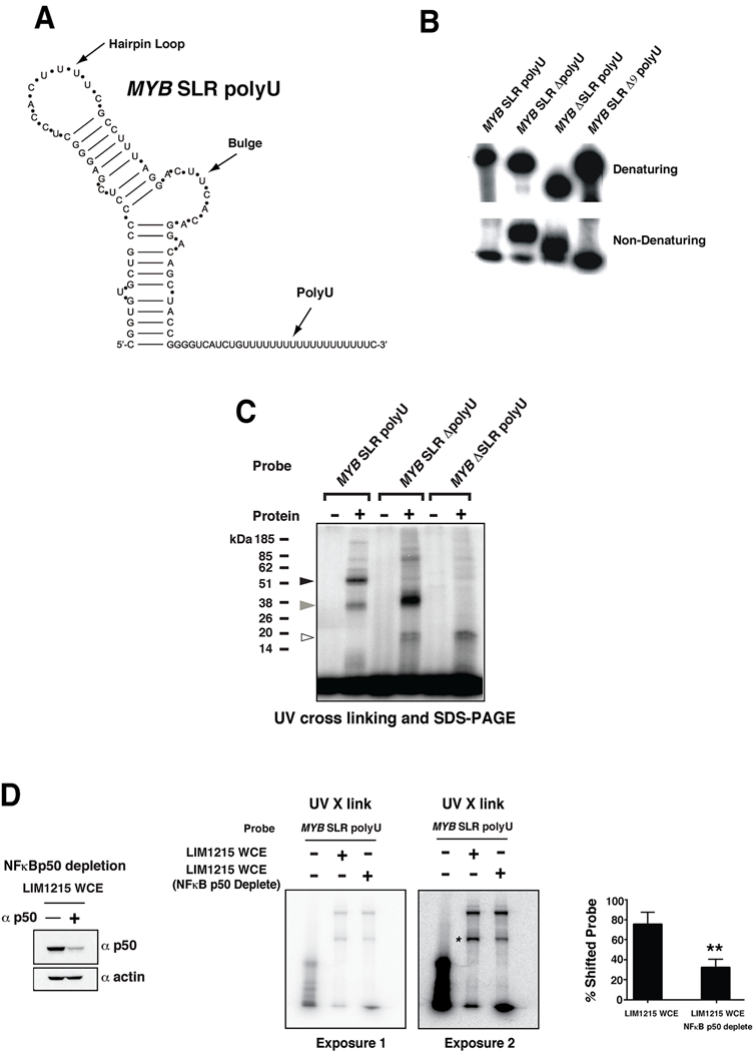
Cellular proteins bind to the *MYB* SLR polyU RNA. (A) *MYB* SLR polyU structure analysis. The mfold predicted structure of the *MYB* SLR polyU is shown. (B) Electrophoretic mobility of radiolabeled *MYB* SLR polyU RNA transcripts (*MYB* SLR polyU; 310 bases), polyU tract-deleted (*MYB* SLR ΔpolyU; 291 bases), stem-loop deleted (*MYB* ΔSLR polyU; 249 bases) and 9 of the 19 polyU residues deleted (*MYB* SLR Δ9 polyU; 300 bases). RNAs were subjected to electrophoresis in a denaturing 4% acrylamide gel where probes migrate according to size and a 4% non-denaturing acrylamide gel where secondary structure is maintained. (C) LIM1215 whole cell extracts were incubated with radiolabeled RNA probes generated from pBluescript II KS *MYB* SLR polyU, polyU tract deleted (*MYB* SLR ΔpolyU) and stem-loop region deleted (*MYB* ΔSLR polyU) templates and subjected to UV-cross linking followed by SDS-PAGE. Two species (~50 kDa, black arrow and ~ 38 kDa, grey arrow) were observed with the *MYB* SLR polyU probe, and one species (~ 38 kDa, grey arrow) with the *MYB* SLR ΔpolyU probe. A ~20 kDa doublet is also evident with the *MYB* ΔSLR polyU and *MYB* SLR ΔpolyU probes, white arrow. (D) LIM1215 whole cell extract was depleted of NFκBp50 by incubation with NFκBp50 antibody. Depletion of NFκBp50 was confirmed by Western blot analysis. Depleted extracts were subsequently incubated with radiolabeled *MYB* SLR polyU RNA probe and subjected to UV-cross linking followed by SDS-PAGE. Two phosphorimaging exposures of the same SDS-PAGE gel are shown to highlight the reduction in the 50kDa signal as indicated by the asterisk. Phosphorimaging quantitation confirmed reduction of the 50kDa signal. Error bars represent mean ± SEM, ** P <0.01.

Soluble protein was extracted from LIM1215 or 293 cells with NP40 lysis buffer (0.64% Nonidet P-40, 5 mM KCl, 2 mM MgCl_2_, 500 mM NaCl, 10 mM Tris, pH8). Soluble protein cell extracts or recombinant proteins were resolved on 10% SDS PAGE gels or 4–12% NuPAGE MOPs gels (Invitrogen) and transferred to PVDF membrane. Membranes were probed with anti-c-Myb1.1 [30], anti-V5 (V5-10; Sigma), anti-NFκBp50 (E10; Santa Cruz, SC), anti-CyclinT1 (T18; SC), anti-CDK9 (L19; SC), anti-HIV-1 Tat (ab42359), anti-NFκBp65 (A: SC), anti-NFκBp65 (C20; SC) or anti-pan-Actin (C4; MP Biomedicals). Membranes were probed with HRP secondary antibodies (Bio-Rad). For the NFκBp50 depletion experiment in Fig 1D, LIM1215 extracts were incubated overnight with anti-NFκBp50 (C19: SC) and NFκBp105 (C19: Cell Signaling). NFκBp50 immunoprecipitates were subsequently removed by incubating extracts with Protein A/G resin (Santa Cruz).

### RT-PCR

Nuclear RNA was isolated from 293 cells transfected with pCMVTat and subjected to RT-PCR to detect intron 1 RNA pre and post-*MYB* SLR attenuator. RT-PCRs used random primers for AMV reverse transcriptase reactions. For Quantitative RT-PCR, RNA was recovered using TRIZOL extraction and RNA reverse transcribed by Superscript III (Invitrogen) and random primers. The cDNA samples were assayed by SYBR-green RT-PCR.

### Nuclear run-on transcription

Nuclear run on assays were performed as described [20, 21]. Transcriptional activity was normalized to GAPDH amplicon and the steady state rate of transcription for each transcript length. Densitometric analysis of the radioactivity bound to the filters was performed using Imagequant software and represents the mean values obtained from duplicate filters.

### *MYB* SLR RNA probes and analysis

Radiolabeled RNA transcripts were prepared using the MEGAshortscript kit (Ambion) and 500 ng of *Eco*RV-linearized pBluescriptIIKS-*MYB* SLR polyU, *MYB* ΔSLR polyU, *MYB* SLR ΔpolyU, *MYB* SLR Δ9 polyU or 500 ng of *Hind* III-linearized pGEM-TAR, bulge mutant *TAR*, *MYB* SLR polyU, *MYB* ΔSLR polyU or *MYB* SLR ΔpolyU and 1 μl [α-^32^P] UTP (3,000 Ci/mmol; Perkin Elmer). Transcription products were precipitated with ethanol and loaded onto a 0.5X TBE/4% PAGE gel. Denaturing electrophoresis used gels containing 6M urea and samples were heated to 95°C in formamide prior to loading. Gels were dried and the radiolabeled RNA detected by Phosphorimager analysis. Mfold version 3.0 was used to determine RNA secondary structures [31]. For RNA EMSAs probes were gel purified from 8M urea polyacrylamide gels.

### RNA and DNA mobility shift assays

RNA EMSA were performed with [^32^P UTP]-labeled RNA probes and recombinant Tat or NFκBp50 protein in reactions containing 30 mM Tris pH8, 70 mM KCl, 12% glycerol, 1.3 mM DTT, 0.01% NP40, 5.5 mM MgCl_2_. For super shift experiments antibody against HIV-1 Tat (ab42359; Abcam) or NFκBp50 (NLS; SC) was added following RNA-protein binding. Reactions were resolved on 0.5X Tris-glycine/5% non-denaturing PAGE (19:1) gels. NFκBp50 DNA EMSAs were performed with ^32^P-labeled IgκB DNA probe [32] and His-NFκBp50 protein in reactions containing 15 mM HEPES-KOH pH7.9, 60 mM KCl, 7.5% glycerol, 0.05% TX100, 50 μg/ml poly [(dI-dC)] and 0.25 mg/ml BSA. Binding reactions were resolved on 0.5X TBE/6% non-denaturing PAGE (29:1) gels. NFκBp65 DNA EMSAs were performed with ^32^P-labeled HIV-1 LTR DNA probe [33] and NFκBp65 protein (Origene) in reactions containing 25 mM HEPES-KOH pH7.9, 0.5mM EDTA, 50 mM NaCl, 5% glycerol, and 1% NP40. Binding reactions were resolved on 0.5X TBE/6% non-denaturing PAGE (29:1) gels.

### UV cross-linking

Binding reactions contained 1 μl ^32^P-labeled RNA transcript, 2–5 μl soluble LIM1215 protein extract or recombinant NFκBp50 in binding buffer containing 20 mM Hepes-NaOH, pH8, 2 mM spermadine, 10 mM MgCl_2_, 100 mM KCl, 1mM DTT, 2 μg poly (dI-dC) and were exposed to 254 nm UV for 10 minutes. Cross-linked samples were treated with T1 RNase and resolved on a 10% SDS PAGE gel.

### Recombinant proteins

HIV-1 Tat (aa 1–101) was obtained from Advanced Bioscience Laboratories, USA. Recombinant NFκBp50 was obtained from Panomics, USA. NFκBp50 RHD (aa 42–365) and its mutants were expressed in BL21(DE3) *E*. *coli* at 30°C, purified with Talon resin and NFκBp50 proteins eluted in 300 mM imidazole. NFκBp50 preparations were dialysed against (50 mM Tris pH8, 100 mM KCl, 10% Glycerol, 0.5 mM EDTA). NFκBp65 was obtained from Origene, USA.

### Quantitation and Statistics

Statistical analysis was calculated using Graphpad Prism version 5.0 (Graphpad Software Inc, USA). Assays were repeated in triplicate to calculate the mean ± SEM unless otherwise stated. Student-T test was used to compare groups.

## Results

### *MYB* SLR RNA has a secondary structure

We modeled the SLR polyU RNA transcribed from intron 1 of *MYB* and showed that this sequence can block elongation (Fig 1A) [20, 21]. To investigate whether the *MYB* SLR polyU encoded an RNA transcript with secondary structure wt (*MYB* SLR polyU), polyU tract-deleted (*MYB* SLR ΔpolyU), SLR deleted (*MYB* ΔSLR polyU) or *MYB* SLR polyU in which 9 of the 19 polyU residues were deleted (*MYB* SLR Δ9 polyU) transcripts were generated and their electrophoretic mobility examined under denaturing and non-denaturing conditions. Despite its larger mass, *MYB* SLR polyU RNA displayed faster mobility under native conditions, consistent with predictions that the *MYB* SLR has a high degree of secondary structure. Removal of the SLR (*MYB* ΔSLR polyU) or the polyU region (*MYB* SLR ΔpolyU), but not partial deletion of the polyU (*MYB* SLR Δ9 polyU) created mixed populations of RNAs under native conditions (Fig 1B), suggesting that the polyU tract may influence secondary structure. Similarly, point mutation changes to the bulge and hairpin loop regions of the SLR resulted in changes in mobility under native conditions S2A Fig mutation in the bulge of the Mfold predicted stem loop (*MYB* SLR 3L mutation) or a mutation in the Mfold predicted hairpin loop (*MYB* SLR 15C1 mutation) slowed the migration of the *MYB* SLR RNA under native conditions and this was more pronounced in the presence of the 15C1 mutation S2 Fig The Mfold predicted conformation of *MYB* SLR 15C1 mutation RNA differed slightly from the *MYB* SLR and *MYB* SLR 3L mutation RNAs, where a larger hairpin loop was predicted S2 Fig Collectively these data suggest that the *MYB* SLR polyU has a secondary structure.

### Cellular factors engage the *MYB* SLR RNA

We sought to identify cellular factors that engage the *MYB* SLR RNA. Given the CRC cell line LIM1215 expresses relatively high levels of *MYB* [20], we reasoned that this cell line might express factors that engage the SLR and regulate *MYB* transcriptional elongation. UV cross-linking analysis was performed with LIM1215 extracts and intronic *MYB* RNA probes containing the SLR and polyU (*MYB* SLR polyU), a deletion of the SLR (*MYB* ΔSLR polyU) or a deletion of the polyU (*MYB* SLR ΔpolyU). Two bands of ~50 and ~38 kDa were resolved when the *MYB* SLR polyU probe was used (Fig 1C). A similarly migrating band of ~38 kDa was observed in the presence of *MYB* SLR ΔpolyU probe. The ~50 kDa band was absent when *MYB* SLR ΔpolyU or *MYB* ΔSLR polyU probes were employed (Fig 1C).

Previous studies have implicated NFκB in the regulation of *MYB* elongation via intron 1 [23–25]. Accordingly we hypothesized that the ~50 kDa binding activity observed in the UV cross-linking experiments represented the interaction of NFκBp50 with the SLR (Fig 1C). In order to directly show that the 50kDa UV cross-linked band was indeed NFκBp50, UV cross-linking experiments were performed with *MYB* SLR polyU RNA probe and LIM1215 protein extracts in which NFκBp50 was depleted. Western blot analysed confirmed depletion of NFκBp50 from the cell extracts (Fig 1D) and formation of the 50kDa UV cross-linked band was reduced two fold in reactions that contained NFκBp50 depleted cell extracts (Fig 1D). Collectively these data suggest that the 50kDa band observed in UV cross-linking experiments represented the binding of NFκBp50 to *MYB* SLR polyU RNA probe.

### NFκBp50 binds the *MYB* SLR RNA

**Fig 2:**
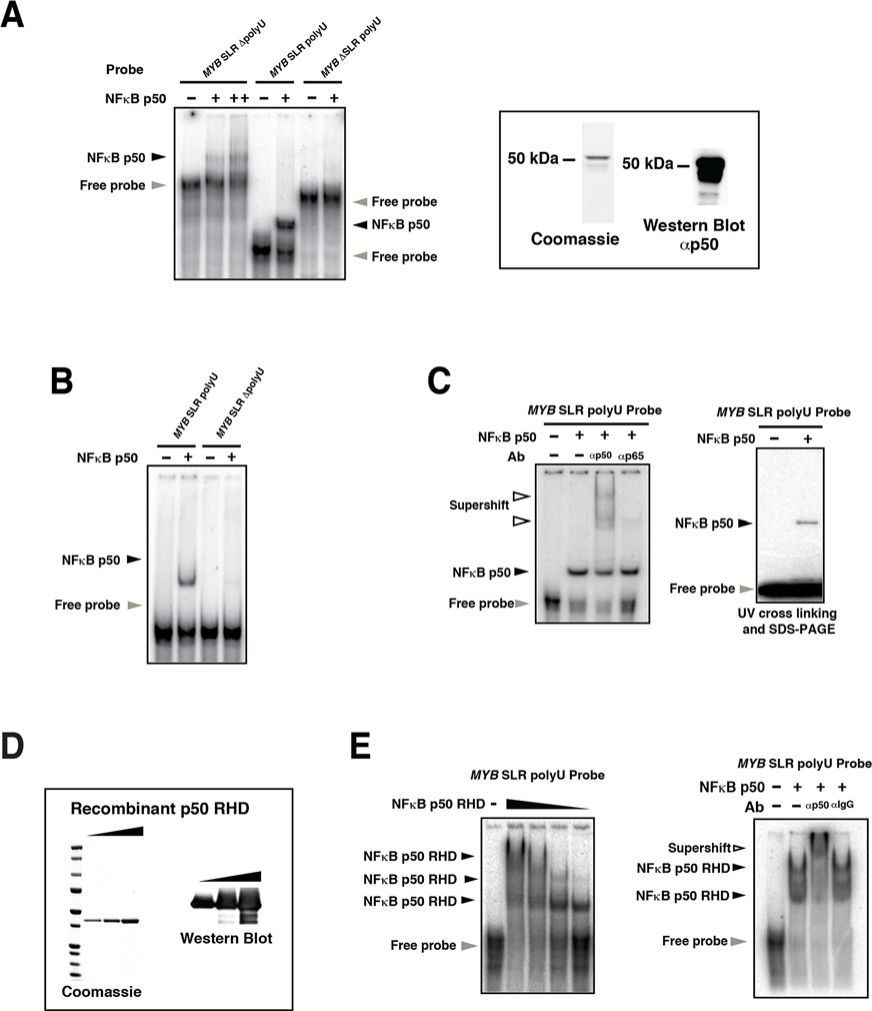
NFκBp50 binds directly to the *MYB* SLR polyU. (A) Radiolabeled RNA probes generated from pBluescript II KS *MYB* SLR polyU, polyU tract-deleted (*MYB* SLR ΔpolyU) or stem-loop region deleted (*MYB* ΔSLR polyU) templates were incubated with 50 ng of NFκBp50 or in the case of the *MYB* SLR ΔpolyU probe with 25 ng and 50 ng NFκB p50 and the reactions resolved on a 5% Tris-glycine gel. Coomassie gel and Western blot analysis of the NFκBp50 protein are shown. (B) Radiolabeled RNA probes generated from pGEM*-MYB* SLR polyU or *MYB* SLR ΔpolyU were incubated with 50 ng of NFκBp50 and the reactions resolved on a 5% Tris-glycine gel. (C) Left; an RNA probe generated from pGEM*-MYB* SLR polyU was incubated with 50 ng of NFκBp50 and NFκBp50-*MYB* SLR polyU RNA-protein complexes were supershifted by the addition of anti-NFκBp50 antibody. Anti-NFκBp65 antibody was used as a control. Right; RNA shifts were performed as above and the reactions subjected to UV-cross linking and SDS-PAGE. (D) NFκBp50 RHD analysed by Coomassie staining and Western blot analysis. (E) Left; an RNA probe generated from pBluescript II KS *MYB* SLR polyU was incubated with increasing amounts of recombinant NFκBp50 RHD and the reactions resolved on a 5% Tris-glycine gel. Right; an RNA probe generated from pBluescript II KS *MYB* SLR polyU was incubated with recombinant NFκBp50 RHD and NFκBp50-*MYB* SLR polyU RNA-protein complexes were super-shifted by the addition of anti-NFκBp50 antibody. Anti-rabbit IgG was used as a control. In (A-E) the black arrows indicate the position of the NFκBp50-*MYB* polyU SLR or NFκBp50*-MYB* SLR ΔpolyU RNA complexes; white arrows show the position of the complexes in the presence of the anti-p50 antibody; grey arrow indicates free *MYB* polyU SLR or *MYB* SLR ΔpolyU RNA probes.

The above experiments suggested that NFκBp50 could bind to the *MYB* SLR RNA. To examine this possibility RNA EMSAs were performed with recombinant NFκBp50 and intronic *MYB* RNA probes *MYB* SLR polyU, *MYB* ΔSLR polyU or *MYB* SLR ΔpolyU. NFκBp50 formed a complex with *MYB* SLR polyU RNA but was unable to shift probe in which the SLR was deleted (*MYB* ΔSLR polyU). Removal of the polyU tract (*MYB* SLR ΔpolyU) substantially reduced NFκBp50 binding (Fig 2A).

In order to establish that NFκBp50 was binding directly to the SLR, EMSAs were performed with shorter RNA probes that encompassed only the SLR and polyU (*MYB* SLR polyU) or only the SLR (*MYB* SLR ΔpolyU) (Fig 2B). NFκBp50 formed a complex with the *MYB* SLR polyU probe (Fig 2B). However, removal of the polyU (*MYB* SLR ΔpolyU) reduced NFκBp50 binding (Fig 2B). Finally, in order to confirm that the complex formed with the *MYB* SLR polyU probe was indeed NFκBp50 we performed EMSAs with *MYB* SLR polyU RNA probe and included NFκBp50 antibody (Fig 2C). NFκBp50 formed a complex with the *MYB* SLR polyU probe that was super-shifted in the presence of the NFκBp50 antibody (Fig 2C). Furthermore, when reactions were subjected to UV cross-linking a single band of 50 kDa was resolved (Fig 2C).

We next assessed whether the Rel homology domain (RHD), responsible for the DNA binding activity of NFκBp50 [32], could engage the *MYB* SLR RNA. Indeed the NFκBp50 RHD (aa 42–365) formed a complex with *MYB* SLR polyU RNA and when increasing amounts of RHD were added the *MYB* SLR polyU RNA probe was moved into multiple complexes that were super-shifted by NFκBp50 antibody (Fig 2D and 2E).

**Fig 3:**
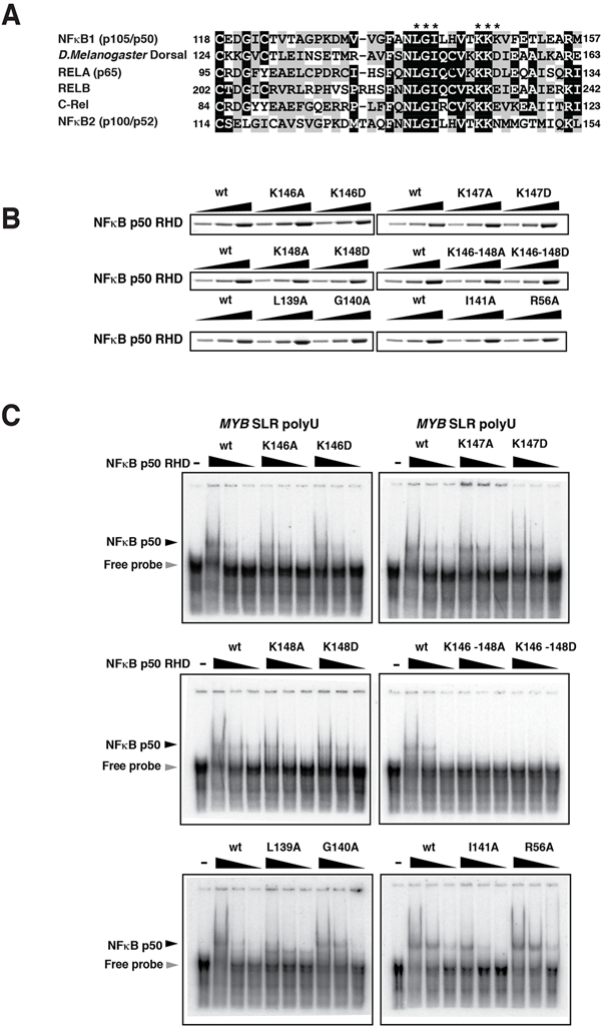
The NFκBp50 RHD binds the *MYB* SLR polyU RNA via a lysine-rich region. (A) Sequence alignment of the RHD of NFκB proteins. Black boxes indicate amino acid identity and grey boxes indicate similarity. The numbers refer to the first position of the segments within the respective proteins. The asterisk refers to residues that were analyzed by mutagenesis. (B) Wild type (wt) and mutant NFκBp50 RHDs analyzed by Coomassie staining. (C) Radiolabeled RNA probe generated from pBluescript II KS *MYB* SLR polyU template was incubated with 12.5 ng of recombinant wt or mutant NFκBp50 RHD as indicated and the reactions resolved on a 5% Tris-glycine gel. The black arrow indicates the position of the NFκBp50 RHD-*MYB* SLR polyU RNA complex; the grey arrow indicates free probe.

Within the RHD we pinpointed two motifs (aa 139–141; LGI) and (aa 146–148; KKK) that were highly conserved amongst the various human and mouse NFκB family members (Fig 3A). Small clusters of highly basic amino acids rich in Lys residues are features of proteins that bind to RNA, with the positive charge of these residues complementing the negative charged surface of RNA [34]. We therefore sought to determine if the KKK residues contributed to the *MYB* SLR polyU RNA binding activity of NFκBp50. NFκBp50 RHD mutants were generated in which the Lys residues were substituted singly or collectively with Ala or Asp (Fig 3B). Single substitution of the Lys residues 146, 147 or 148 subtly reduced NFκBp50 RHD binding to *MYB* SLR polyU RNA (Fig 3C). In contrast, binding of p50 RHD was substantially reduced by substituting all three Lys for Ala or Asp (Fig 3C). The LGI residues were also each substituted for Ala (Fig 3B). Substituting the Leu or Ile residues reduced NFκBp50 binding to *MYB* SLR polyU RNA probe while substituting the Gly had little effect (Fig 3C).

To determine if the KKK and LGI motifs represented a modules that only bound RNA or whether they could also contribute to the DNA binding activity of NFκBp50, EMSAs were performed with the NFκBp50 RHD mutants and an IgGκB DNA probe [32]. Single or collective substitution of the Lys residues for Ala or Asp reduced binding of NFκBp50 RHD to IgGκB DNA S3 Fig Substituting the Leu, Ile or Gly residues for Ala reduced NFκBp50 RHD binding and the effect of these mutations mirrored that observed for the DNA-binding residues Arg 56 and 58, Tyr 59, Glu 62 and His 66 substituted for Ala [35] and S3 Fig We also observed that substituting Arg 56 for Ala had no effect on RNA binding (Fig 3C). These results indicate that NFκBp50 shares conserved sequences (aa 139–141; LGI) and (aa 146–148; KKK) that contribute to the DNA and RNA binding activity of NFκBp50.

### NFκBp50 and p65 induce transcriptional elongation via the *MYB* SLR

**Fig 4:**
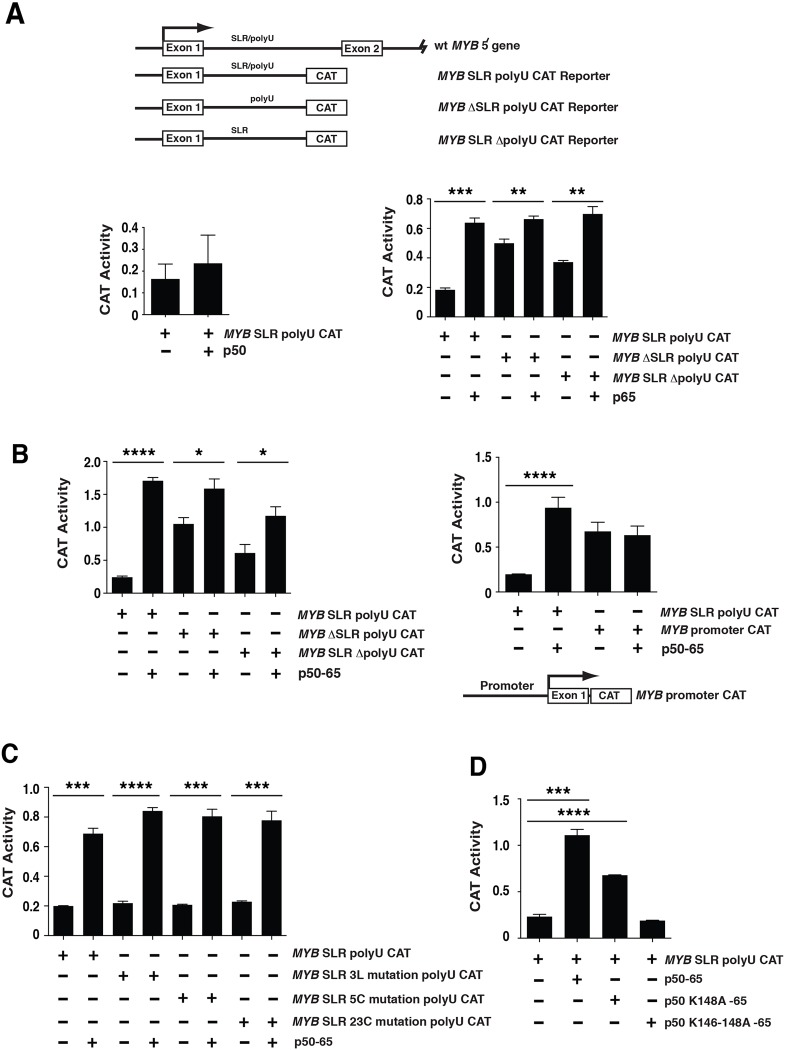
NFκBp50 and NFκBp65 induce *MYB* elongation via the *MYB* SLR polyU. (A) Top panel: The 5´ genomic structure of *MYB* and the *CAT* reporter constructs is depicted. *MYB* ΔSLR polyU CAT has a 76 bp deletion of the SLR sequence up to the 19 nucleotide polyU stretch. *MYB* SLR ΔpolyU CAT contains a deletion of the 19 nucleotide polyU stretch. Bottom left panel: Transactivation studies in 293 cells using 2 μg of the *MYB* SLR polyU CAT reporter and 0.5 μg of pcDNA NFκBp50. Bottom right panel: Transactivation studies in 293 cells using 2 μg of the *MYB* SLR polyU CAT, *MYB* ΔSLR polyU CAT or *MYB* SLR ΔpolyU CAT reporters and 0.5 μg of pcDNA NFκBp65. (B) Left panel: Transactivation studies in 293 cells using 2 μg of the *MYB* SLR polyU CAT, *MYB* ΔSLR polyU CAT or *MYB* SLR ΔpolyU CAT reporters and 0.25 μg of pcDNA NFκBp50-p65; Right panel: Transactivation studies in 293 cells using 2 μg of the *MYB* SLR polyU CAT or *MYB* Promoter CAT reporters and 0.25 μg of pcDNA NFκBp50-p65. (C) Transactivation studies in 293 cells using 2 μg of the *MYB* SLR polyU CAT, *MYB* SLR 3L mutation polyU CAT, *MYB* SLR 5C mutation polyU CAT or *MYB* SLR 23C mutation polyU CAT reporters with 0.25 μg of pcDNA NFκBp50-p65. (D) Transactivation studies in 293 cells using 2 μg of the *MYB* SLR polyU CAT reporter with 0.25 μg of pcDNA NFκBp50-p65, 0.25 μg of pcDNA NFκBp50 K148A-p65 or 1 μg of pcDNA NFκBp50 K146-148A-p65. Error bars represent mean ± SEM, * P <0.05, ** P <0.01, *** P <0.001, **** P <0.0001.

The above experiments suggested that NFκBp50 might engage the *MYB* SLR polyU to regulate *MYB* elongation. To examine this possibility, the potential of NFκBp50 to regulate elongation in gene reporter assays was assessed using a series of CAT reporter constructs containing the promoter, exon 1 and intron 1 with or without the *MYB* SLR or polyU [21] (Fig 4A).

NFκBp50 did not stimulate transcription from the *MYB* SLR polyU elongation CAT construct (Fig 4A). In contrast transfected NFκBp65 stimulated transcription in a *MYB* SLR and polyU tract dependent manner (Fig 4A). Furthermore, consistent with previous published results [21], the activity of *MYB* ΔSLR polyU CAT and *MYB* SLR ΔpolyU CAT was higher than *MYB* SLR polyU CAT, confirming that the *MYB* SLR and polyU sequences negatively impacted on *MYB* transcriptional activity (Fig 4A). Because of these observations we tested whether NFκBp65 could associate with the *MYB* SLR polyU in RNA EMSA experiments. However, we did not detect a robust interaction between NFκBp65 and *MYB* SLR polyU RNA S4 Fig Furthermore, we confirmed the nucleic acid binding activity of the recombinant NFκBp65 preparation by performing DNA EMSAs with an HIV-1 LTR radiolabeled probe previously shown to support binding of NFκBp65 [33]. Our DNA EMSA results also showed that recombinant NFκBp65 could bind DNA and promote binding of NFκBp50, under conditions where the concentration of NFκBp50 was limiting S4 Fig

The ability of NFκBp65 to activate the *MYB* SLR polyU CAT but not directly bind *MYB* SLR polyU RNA in vitro led us to conclude that the induction of *MYB* SLR polyU elongation by NFκBp65 may reflect a mechanism in which NFκBp65 is recruited to the *MYB* SLR polyU through its direct interaction with NFκBp50 that is bound to the *MYB* SLR thus forming an NFκBp50-65 heterodimer on the *MYB* SLR polyU. Indeed it is well established that NFκBp65 binds DNA inefficiently and that the NFκBp50-65 heterodimer is the most abundant form of the NFκB dimers that stimulate transcription [32, 36]. To this end we performed RNA EMSA experiments in which NFκBp65 was titrated into NFκBp50-*MYB* SLR polyU binding reactions S4 Fig Under these binding conditions we did not observe additional bands that were indicative of a NFκBp50-65 heterodimer S4 Fig

We next sought to further explore the scenario of a NFκBp50 and p65 complex on the *MYB* SLR polyU by exploiting a functional NFκBp50-65 heterodimeric fusion protein [29]. The NFκBp50-65 chimeric protein contains the RHD of NFκBp50 and the C-terminal activation domain (AD) of NFκBp65 [29]. The NFκBp50-65 fusion induced a dramatic stimulation of *MYB* SLR polyU CAT transcription in a SLR and polyU tract-dependent manner (Fig 4B). In contrast the NFκBp50-65 fusion failed to induce *MYB* promoter activity from the *MYB* construct containing only the *MYB* proximal promoter and exon 1 sequence (*MYB* promoter CAT) (Fig 4B). We also examined NFκBp50-65 regulation through the *MYB* SLR using *MYB* SLR polyU CAT constructs containing point mutations predicted to disrupt the *MYB* SLR structure, i.e. *MYB* SLR 3L, 5C or 23C mutation polyU CAT (Fig 4C). In each case NFκB p50-65 mediated stimulation of *MYB* SLR polyU CAT was not impacted on by the *MYB* SLR mutations (Fig 4C). Collectively these data show that the NFκBp50-65 chimera mediated its effect on *MYB* elongation through the *MYB* SLR polyU sequence and that the polyU was a major determinant regulating this activity.

We next examined whether the conserved lysine sequence within the NFκBp50 RHD (aa 146–148 (Fig 3) influenced the effect of the NFκBp50-65 fusion on *MYB* elongation. Lysine mutations that disrupted both the RNA and DNA binding activity of NFκBp50 (K146-148A) (Fig 3C) and S3 Fig reduced the stimulation of *MYB* elongation by the p50-65 chimera (Fig 4D). The K148A substitution that modestly reduced the RNA binding activity, but not the DNA binding activity, of NFκBp50 (Fig 3C) and S3 Fig also reduced the NFκBp50-65 mediated stimulation of *MYB* SLR polyU elongation (Fig 4D). Importantly, we also observed that mutation of the DNA binding reside Arg 56 also reduced the NFκBp50-65 mediated stimulation of *MYB* SLR polyU elongation S3 Fig Together these data suggest that the nucleic acid binding activity of NFκBp50-65 that includes its RNA and DNA binding functions, mediates stimulation of *MYB* SLR polyU CAT elongation.

**Fig 5:**
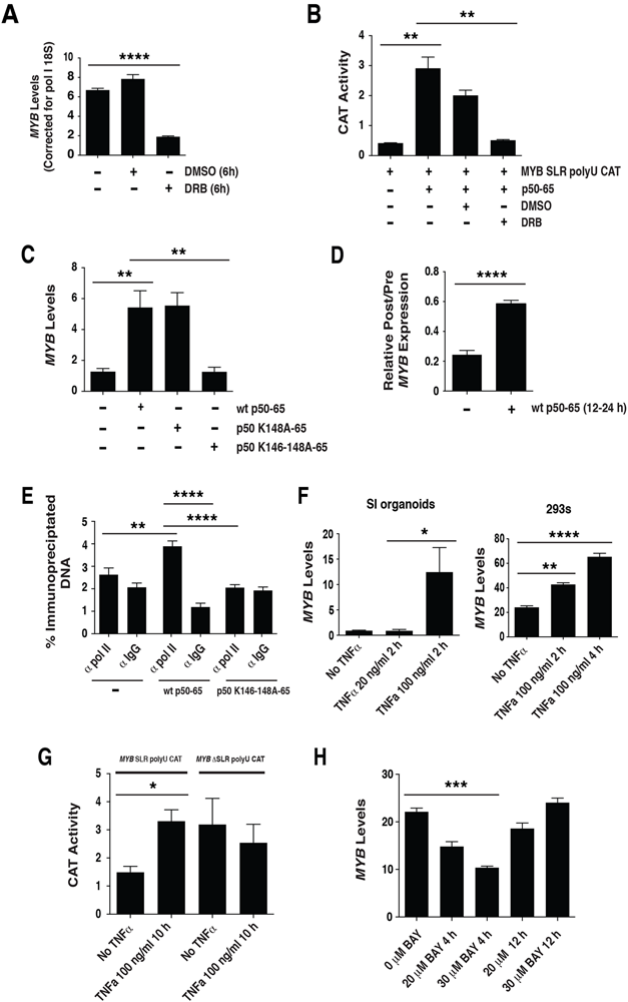
NFκBp50-p65, P-TEFb and TNFα influence *MYB* elongation. (A) 293 cells were treated with the P-TEFb inhibitor DRB for 6 h and endogenous *MYB* expression assessed by QPCR. (B) Transactivation studies in 293 cells using 2 μg of the *MYB* SLR polyU CAT reporter and 0.125 μg of pcDNA NFκBp50-p65. At 12 h post transfection cells were treated with DRB and incubated for a further 24 h. (C) NFκBp50-p65 induces endogenous *MYB*. Total RNA was isolated from 293 cells transfected with; 1 μg of pcDNA NFκB p50-p65, 1 μg of pcDNA NFκBp50 K148A-p65 or 4 μg of pcDNA NFκBp50 K146-148A-p65 and analyzed by Q-PCR to measure *MYB* expression levels and (D) 1 μg of pcDNA NFκB p50-p65 and analyzed by Q-PCR to measure intronic pre-mRNA *MYB* transcript upstream (preSLR) and downstream (post SLR) of the *MYB* SLR polyU. Data are expressed as a ratio “post/pre”, a measure of the amount of transcription through the SLR. (E) ChIP analysis of RNA polymerase II levels at the *MYB* SLR polyU. 293 cells were transfected with pcDNA, NFκB p50-p65 or pcDNA NFκBp50 K146-148A-p65. Cross-linked chromatin extracts were prepared at 48h post transfection and RNA polymerase II was detected by anti-pol II followed by Q-PCR. (F) SI organoids cultures and 293 cells were exposed to TNFα (20–100 ng/ml) for the times indicated and total RNA was isolated and analyzed by Q-PCR to measure *MYB* expression levels. (G) 293 cells were transfected with 2 μg of the *MYB* SLR polyU CAT or *MYB* ΔSLR polyU CAT reporter. At 24 h post transfection cells were exposed to 100 ng/ml TNFα for 10 h and CAT reporter activity assessed. (H) 293 cells were exposed to BAY inhibitor for the times indicated and total RNA was isolated and analyzed by Q-PCR to measure *MYB* expression levels. Error bars represent mean ± SEM, * P <0.05, ** P <0.01, *** P <0.001, **** P <0.0001.

We next examined whether the elongation factor P-TEFb influenced *MYB* expression. Previous studies have established that the P-TEFb inhibitory drug DRB inhibits elongation that leads to the synthesis of longer *MYB* transcripts [22]. We therefore treated 293 cells with DRB for 6 h and assessed the expression of *MYB* by QPCR. *MYB* expression was significantly down regulated in drug treated cells (Fig 5A). Furthermore, DRB treatment of 293 cells inhibited the NFκBp50-65 mediated induction of *MYB* elongation from the *MYB* SLR polyU CAT reporter (Fig 5B). Collectively these data suggest that P-TEFb could influence NFκBp50-65 mediated induction of *MYB* elongation.

### NFκBp50 and p65 induce *MYB* expression

Having established a relationship between NFκBp50, p65 and *MYB* SLR polyU transcriptional elongation, we next sought to determine whether these factors could influence endogenous *MYB* expression. To this end the NFκBp50-65 chimera was transfected into 293 cells and *MYB* expression assessed. Q-RT-PCR analysis demonstrated that endogenous *MYB* levels were increased upon transfection of cells with NFκBp50-65 (Fig 5C). Conversely, reduced induction of *MYB* was observed using NFκBp50-65 containing Lys mutations that disrupted both the RNA and/or DNA binding activity of NFκBp50-65 (K146-148A) (Fig 5C) and NFκBp50-65 (R56A) S3 Fig However, in this context the K148A alone substitution did not effect NFκBp50-65 mediated stimulation of *MYB* SLR polyU elongation (Fig 5C). In addition, NFκBp50-65 enhanced the synthesis of intronic pre-mRNA *MYB* transcript past the *MYB* SLR polyU (post SLR) indicating that NFκB p50-p65 could relieve the transcriptional attenuation of *MYB* (Fig 5D). Consistent with this finding we observed by chromatin immunopreciptation (ChIP) analysis an increase in the enrichment of RNA polymerase II at the *MYB* SLR poly U in the presence of NFκBp50-65 (Fig 5E).

We next determined whether endogenous NFκBp50 and p65 could induce *MYB* expression *in vivo*. Previous studies have established that NFκB pathway can be activated by the pro-inflammatory cytokine TNFα [37]. Q-RT-PCR analysis demonstrated that endogenous *MYB* levels were increased upon exposure of small intestinal organoid cultures and 293 cells to TNFα (Fig 5F). Similarly, *MYB* SLR polyU CAT activity was stimulated when 293 cells were treated with TNFα and when the SLR was removed (*MYB* ΔSLR polyU CAT) the addition of TNFα did not induce reporter activity (Fig 5G). Furthermore, inhibition of the NFκB pathway with the BAY inhibitor BAY 11–7082 reduced endogenous *MYB* levels in a time dependent manner (Fig 5H). Taken together, the above data indicate that the NFκB factors p50 and p65 can induce *MYB* expression via the intron 1 *MYB* SLR polyU.

### Tat induces *MYB* transcriptional elongation

**Fig 6:**
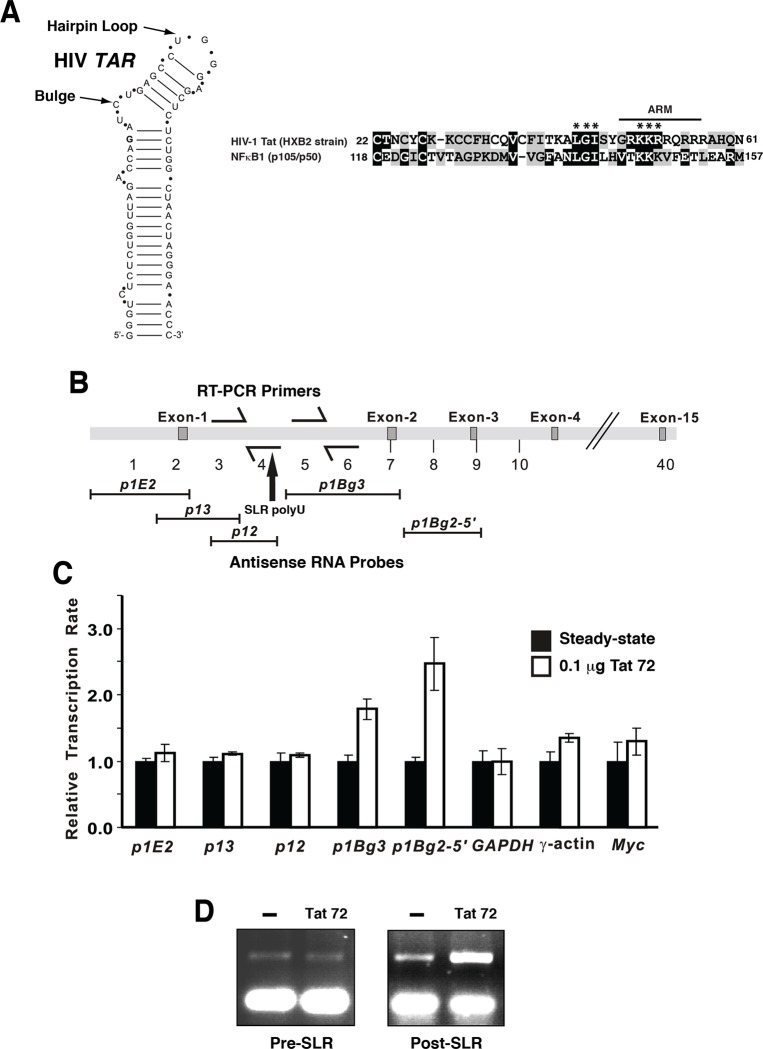
HIV-Tat induces *MYB* expression and transcriptional elongation. (A) The RNA stem loop structure of the HIV *TAR* [26]. Sequence alignment of the *TAR* binding region of HIV-1 Tat with the RHD of NFκBp50. Black boxes indicate amino acid identity and grey boxes indicate similarity. The Tat RNA binding domain (ARM motif) is indicated by the black line. The numbers refer to the first position of the segments within the respective proteins. The asterisk refers to NFκBp50 residues that were analyzed by mutagenesis. (B) The genomic arrangement of the *MYB* locus. The anti-sense RNA probes, the location of the SLR region within intron 1 and the location of RT-PCR primers used to examine RNA elongation across this region by nuclear run-on transcription are shown. (C) Transcription of the *MYB* gene in 293 cells as assessed by nuclear run-on transcription. Nuclear run on assays were performed as described [20, 21]. Transcriptional activity was normalized to GAPDH signal and the steady state rate of transcription for each transcript length. Relative transcription between untransfected cells and cell transfected with Tat is shown. Densitometric analysis of the radioactivity bound to the filters was performed using Imagequant software and represents the mean values obtained from duplicate filters. (D) Nuclear RNA was isolated from 293 cells transfected with 100 ng pCMVTat (72) and subjected to RT-PCR to detect intron 1 RNA pre- and post-*MYB* SLR attenuator region.

Comparative sequence alignment revealed that residues (aa118-157) of the NFκBp50 RHD were similar to a highly conserved basic region of Tat (aa 48–57), which functions as an RNA binding domain (Arg rich motif; ARM) [26, 38] (Fig 6A). The NFκBp50 RHD shares two Lys residues (KK:aa 146–147) with the ARM of Tat that are important for *TAR* RNA binding [39]. A third Lys residue (K:aa 148) was a conserved change. Upstream of the ARM, Tat and NFκBp50 also shared Leu, Gly and Ile residues (LGI: aa 139–141) (Fig 6A). This region of Tat is required for its transactivation function and is part of a sequence required for interaction with CyclinT1 [40, 41].

Because of the similarities between NFκBp50 RHD and the Tat RNA binding domain we reasoned that HIV-Tat might also influence endogenous *MYB* expression via elongation. To examine this possibility, nuclear run-on assays were performed on 293 cells expressing Tat. Probes 3´ to the SLR region showed increased signal in the presence of exogenous expressed Tat suggesting that Tat increased elongation into regions downstream of the SLR (Fig 6B and 6C). Furthermore, in the presence of exogenous expressed Tat the level of post-SLR RNA was higher (Fig 6D).

**Fig 7:**
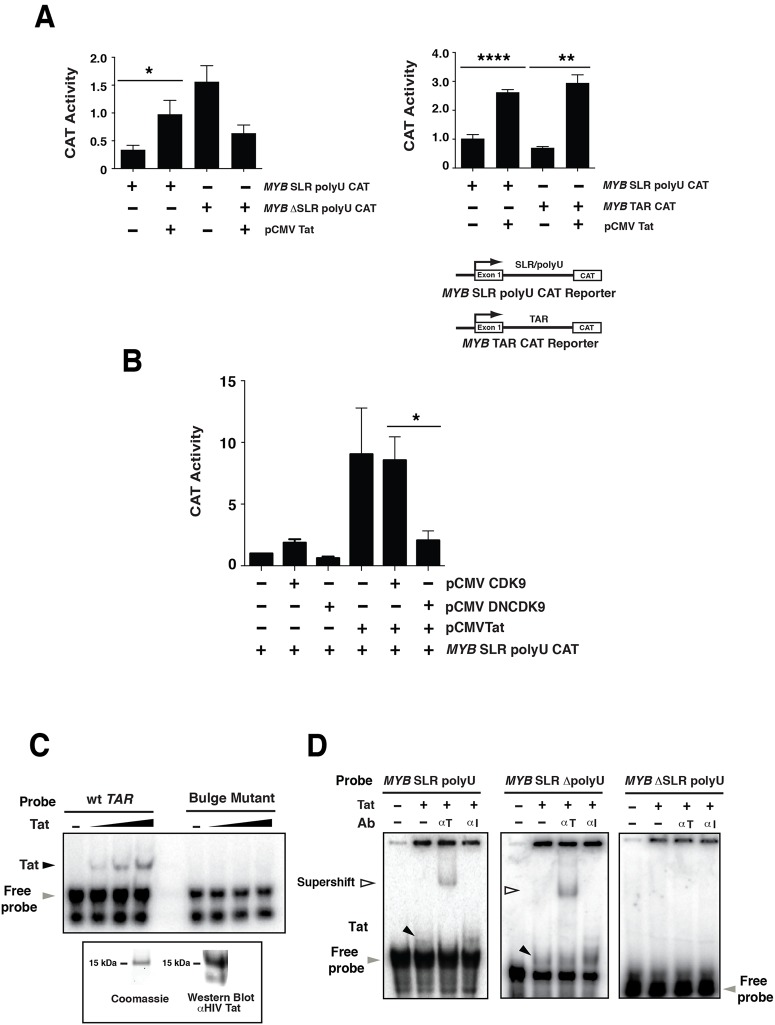
HIV-Tat increases *MYB* reporter activity via the intron 1 *MYB* SLR. (A) Transactivation studies in 293 cells using: Left panel, 2 μg of *MYB* SLR polyU CAT or *MYB* ΔSLR polyU CAT and 2 μg of pCMV Tat (101). Right panel, 2 μg of *MYB* SLR polyU CAT or *MYB* TAR CAT and 2 μg of pCMV Tat (101). (B) Transactivation studies in 293 cells using 2 μg of *MYB* SLR polyU CAT reporter, 1.5 μg pCMV Tat (72) and 1.5 μg of pCMVCDK9 or pCMVDNCDK9. (C) *TAR* or bulge mutant (T+23A) *TAR* RNA probes were incubated with 25, 50 and 100 ng of Tat and reactions resolved on a 5% Tris-glycine gel. The black arrow indicates the position of the Tat-*TAR* RNA complex; the grey arrow indicates free *TAR* RNA probe. Coomassie gel and Western blot analysis confirming the expression and integrity of Tat is shown. (D) Binding of Tat to the *MYB* SLR polyU. RNA probes were generated from *pGEM-3Zf MYB* SLR polyU, *MYB* SLR ΔpolyU or *MYB* ΔSLR polyU templates, incubated with 50 ng of Tat and reactions resolved on a 5% Tris-glycine gel. Tat-*MYB* SLR polyU or Tat-*MYB* SLR RNA-protein complexes were super-shifted by the addition of anti-Tat antibody (100 ng). A mouse isotype control IgG (100 ng) was used as a control. The black arrows indicate the position of the Tat-*MYB* SLR polyU or Tat-*MYB* SLR RNA-protein complexes; the white arrows show the position of this complex in the presence of an anti-Tat antibody; the grey arrow indicates free *MYB* SLR polyU, *MYB* SLR ΔpolyU or *MYB* ΔSLR polyU probe. Error bars represent mean ± SEM, * P <0.05, ** P <0.01, **** P <0.0001.

When *MYB* SLR polyU CAT or *MYB* SLR ΔpolyU CAT was co-transfected with Tat an induction of CAT activity was observed (Fig 7A). In contrast Tat failed to induce *MYB* ΔSLR polyU CAT reporter activity, instead reducing activity below basal levels (Fig 7A). Together these CAT reporter data suggested that Tat influenced *MYB* expression and elongation via the *MYB* SLR.

We next examined whether the *MYB* SLR and TAR where inter-changeable. A CAT reporter construct in which the *MYB* SLR was replaced with the HIV *TAR* (*MYB* TAR CAT) (Fig 7A) was co-transfected with Tat and in this context an induction of CAT activity was observed (Fig 7A). Together these data suggest that in the context of the *MYB* intron 1, the SLR and HIV *TAR* are inter-changeable with respect to Tat mediated induction of *MYB*.

Tat recruits CyclinT1 and CDK9 to promote elongation paused at the *TAR* RNA [42]. To determine whether the effects of Tat on *MYB* SLR elongation were influenced by CDK9, 293 cells were co-transfected with *MYB* SLR polyU CAT and Tat as well as CDK9 or a CDK9 dominant negative (DN) form. When *MYB* SLR polyU CAT was co-transfected with CDK9 a slight induction of CAT activity was observed (Fig 7B). Expression of CDK9 did not significantly enhance Tat mediated induction of *MYB* SLR polyU CAT. In contrast, expression of the DNCDK9 negated Tat-mediated activation of *MYB* SLR polyU CAT (Fig 7B). Together these CAT reporter data suggested that CDK9 can influence Tat mediated induction of *MYB* SLR polyU CAT.

### Tat binds the *MYB* SLR

The above experiments suggested that Tat, like NFκBp50, influenced *MYB* expression and elongation via direct binding to the *MYB* SLR. To examine this possibility EMSAs were conducted with recombinant Tat and an RNA probe comprising the SLR and polyU tract (*MYB* SLR polyU). The RNA binding activity of Tat was confirmed by EMSAs in which Tat bound HIV-1 *TAR* RNA (wt TAR) but not *TAR* RNA containing a mutation in the Tat binding region [28] (Bulge mutant) (Fig 7C). Under identical binding conditions Tat formed a complex with the *MYB* SLR polyU RNA that was super-shifted by an anti-Tat antibody (Fig 7D). To establish that Tat was binding directly to the SLR sequence, gel shifts were also performed with RNA probes that contained a deletion of the polyU (*MYB* SLR ΔpolyU) or a deletion of the SLR (*MYB* ΔSLR polyU) (Fig 7D). Tat bound to *MYB* SLR ΔpolyU RNA but not to the *MYB* ΔSLR polyU RNA (Fig 7D). These observations were consistent with the capacity of Tat to induce *MYB* SLR ΔpolyU CAT activity but not *MYB* ΔSLR polyU CAT activity (Fig 7A).

## Discussion

Many eukaryotic genes are regulated in part by transcriptional pausing [8, 9, 11, 12, 43, 44]. *MYB* is one such gene and an attenuation region responsible for pausing has been mapped within the first intron in both human and mouse genes [18, 20, 21, 23]. Two views are held regarding the pausing mechanism that operates via the intronic attenuation sequences; one dependent upon the DNA template itself and the other employing an RNA stem-loop region (SLR) [19–21, 23–25, 45]. In this study we provide evidence that the nucleic acid binding activity of NFκBp50 that includes its RNA and DNA binding functions can regulate *MYB* via the SLR polyU region.

Several transcription factors regulate transcription by binding both DNA and RNA sequences [46]. Our analysis adds to this list by confirming that NFκBp50 is an RNA binding factor and for the first time suggest that *MYB* transcript is an RNA target for NFκBp50. Collectively our observations also imply that *MYB* is a direct target of the NFκB signaling pathway. Consistent with this view, recent studies have demonstrated that in EBV-infected nasopharyngeal carcinoma cell lines, NFκB signaling regulates *MYB* expression [47]. While the focus here has been on the interaction of NFκBp50 with the *MYB* SLR polyU, of relevance to this study is the possibility that NFκB p50 may also bind HIV *TAR* RNA. In CAT reporter assays we observed that the *MYB* TAR CAT reporter was responsive to NFκB p50-65 and in band shift assays recombinant NFκB p50 shifted a TAR RNA probe S5 Fig In support of these data are recent structure based analyses, which have shown that NFκBp50 is capable of binding TAR RNA [48]. Collectively these observations provide a strong case that NFκBp50 can act as an RNA binding factor.

Previous analysis used MFold to predict the potential secondary structure of the *MYB* SLR polyU [21]. We have now extended this analysis by examining the electrophoretic mobility of *MYB* SLR polyU RNA versus a panel point mutants. Our results indicate that mutation of the SLR region alters its mobility under native conditions, consistent with the prediction that this region forms a secondary structure. Furthermore, our data suggest that the polyU region may play a previously unrecognized role in influencing the formation of the *MYB* SLR secondary structure.

We provide several observations that demonstrate NFκBp50-mediated regulation of *MYB* elongation by an RNA dependent mechanism. First, our UV cross-linking experiments isolated a 50kDa *MYB* SLR polyU RNA binding activity in colon carcinoma cell line LIM1215 extracts. Furthermore, depletion of NFκBp50 from LIM1215 extracts saw a decrease in the level of 50kDa signal in UV cross-linking experiments. Second, in gel shift experiments purified recombinant NFκBp50 bound to a *MYB* SLR polyU RNA probe. Third, reporter assays demonstrated a role for NFκBp50 in relieving the transcriptional elongation block through *MYB* intron 1 in an SLR- and CDK9-dependent manner. This activity was dependent on the nucleic acid binding activity of NFκBp50 that includes both its RNA and DNA binding activity. Finally, over-expression of TNFα and BAY inhibitor studies indicated that NFκBp50-p65 could induce endogenous *MYB* expression and enhance the synthesis of intronic pre-mRNA *MYB* transcript past the *MYB* SLR polyU tract (post SLR). An RNA binding activity for NFκBp50 was also underscored by comparative sequence alignments in which residues (aa118-157) of the NFκBp50 RHD were similar to a highly conserved basic region of Tat (aa 48–57), which functions as an RNA binding domain [26, 38]. In this context we demonstrated that Tat could induce the expression and transcriptional elongation of *MYB* and interact directly with *MYB* SLR polyU RNA.

**Fig 8:**
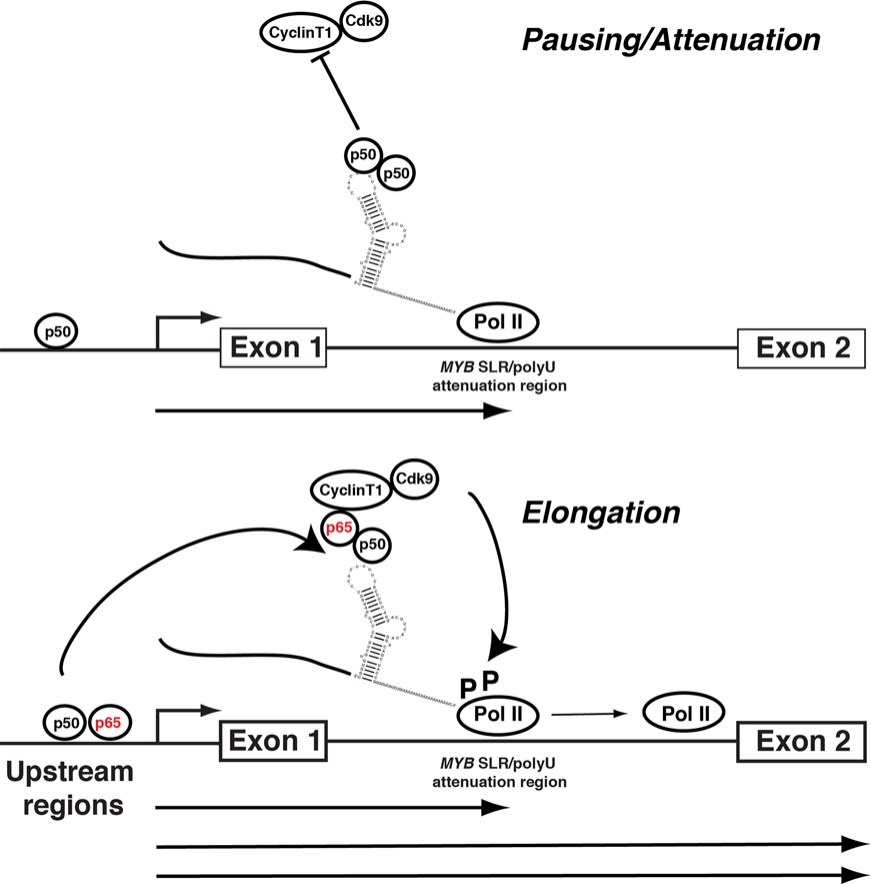
Model for the NFκBp50 and p65 regulation of *MYB* elongation through the intron 1 *MYB* SLR polyU region and upstream sequences. NFκBp50 binding at upstream sequences stimulates transcription from the multiple start sites. Transcripts are then paused/attenuated at the downstream *MYB* SLR polyU. In the absence of NFκBp65, NFκBp50 occupies the *MYB* SLR polyU region and *MYB* transcription is paused. In contrast the formation of a NFκBp50-p65 heterodimer on the *MYB* SLR polyU contributes to the stimulation of *MYB* transcription elongation by the NFκBp65-mediated recruitment of P-TEFb and the subsequent Ser2 phosphorylation of Pol II CTD (elongating form) by CDK9. This model is consistent with i) previous findings that NFκBp65 mediates transcriptional elongation through the direct recruitment of P-TEFb [50] and ii) our recent observations that ERα recruits P-TEFb to a region near the *MYB* SLR polyU and that this interaction is concordant with the accumulation of Ser2 CTD phosphorylated pol II bound to this region and the relief of *MYB* transcriptional attenuation [22]. This model also raises the possibility that the regulation of *MYB* may also involve the interaction of upstream bound NFκBp50/65 with the downstream intron 1 *MYB* SLR polyU. Recent data indicate that enhancer elements loop towards murine *Myb* intron 1 to bring the transcription apparatus to the vicinity of the pausing region and regulate *Myb* attenuation/elongation [19] and we have shown that the upstream regions of *MYB* lie in proximity to the *MYB* SLR polyU S5 Fig Furthermore, recent structure based analyses of HIV-1 LTR interactions suggest that pre-formed NFκBp50-DNA complexes can interact with downstream HIV TAR RNA [48].

Previous studies have implicated NFκB family members in the regulation of transcriptional elongation through intron 1 of the murine *MYB* gene [23–25]. Notably, an NFκB enhancer element resides within intron 1 that is capable of binding NFκBp50, RelA, RelB and c-Rel *in vitro* [23–25]. In these studies, binding of NFκB complexes to this region was linked to the activation of endogenous *MYB* or *MYB*-CAT expression, suggesting that in case of the murine *MYB* gene transcriptional elongation through intron 1 is mediated by a DNA-dependent mechanism. Although the NFκB enhancer is potentially important to the regulation of the endogenous gene, it resides approximately 1.2 kbp downstream of the region investigated here and was excluded from the reporters examined in the current study. Nevertheless, this suggests that NFκBp50 may also contribute to the regulation of *MYB* via a region(s) upstream of the *MYB* SLR polyU. Consistent with this observation other reports have shown a role for a NFκB site found in the *MYB* promoter [49]. Collectively these observations suggest that NFκBp50 may regulate *MYB* through multiple regions/sites within the gene. In this case NFκBp50 bound at upstream sequences may stimulate transcription from the multiple start sites upstream of exon 1. Transcripts that are then stalled at the downstream *MYB* SLR polyU are released by the action of NFκBp50 at the *MYB* SLR polyU. In this way NFκBp50 may regulate *MYB* at at least two points within the gene using its nucleic acid binding activity (Fig 8).

This model of NFκBp50 activity on the *MYB* gene raises the interesting possibility that upstream bound NFκBp50 may also interact with the intron 1 based *MYB* SLR polyU (Fig 8). Indeed recent data indicate that upstream enhancer elements loop towards murine *Myb* intron 1 to bring the transcription apparatus to the vicinity of the pausing region and regulate *Myb* attenuation/elongation [19]. These observations are consistent with data from chromosome conformation capture (3C) assays that we have performed using human Colo201 cells. We found that regions upstream of the *MYB* proximal promoter lie in proximity to the intron 1 *MYB* SLR polyU S5 Fig This effect was lost when Colo201 cells were induced to differentiate with sodium butyrate, concomitant with the downregulation of *MYB* S5 Fig Importantly, the downregulation of Myb is an early event in differentiation that we have previously associated with transcriptional attenuation in intron 1 [20]. The interaction of NFκBp50 bound to upstream DNA regions with downstream RNA elements is supported by recent structure based analyses of HIV-1 LTR interactions in which pre-formed NFκBp50-DNA complexes where found to interact with downstream HIV TAR RNA [48]. We also note recent data in which Tat preassembled at the HIV promoter in a P-TEFb:7SK snRBP complex is able to engage and transition to downstream TAR RNA to regulate P-TEFb mediated stimulation of transcription elongation [51].

We have mapped amino acids 118–157 within the RHD of NFκBp50 as constituting a *MYB* SLR polyU binding domain. Within this region we have pinpointed two motifs (aa 139–141; LGI) and (aa 146–148; KKK) required for the association of NFκBp50 with *MYB* SLR polyU RNA and are highly conserved amongst the various human and mouse NFκB family members. Remarkably, the 118-157aa segment bears a striking similarity with the RNA binding motif of Tat, particularly across the LGI and KKK motifs. Small clusters of highly basic amino acids rich in Arg or Lys residues are features of proteins that bind to RNA, with the positive charge of these residues complementing the negative charged surface of RNA [34]. These sequence features of NFκBp50, coupled with the observations described above reinforce the notion that NFκBp50 can function as an RNA binding factor.

The finding that NFκBp50 is an RNA binding protein is consistent with previous studies that have analyzed the potential of NFκBp50 to bind *in vitro* selected RNA ligands (aptamers) [52–54]. Characterization of an optimal NFκBp50 binding RNA aptamer revealed a highly pre-structured RNA, folded as stem-loop with an asymmetric internal loop [55]. However, the structure of the *MYB* SLR polyU described here differs with little or no sequence similarity with the RNA aptamer. The crystal structure of the NFκBp50-aptamer complex reveals a conserved protein-nucleic acid interface in which the RNA aptamer mimics the κB DNA consensus element by contacting amino acids located in loop 1 and 2 that are involved in DNA binding [56]. Similarly, our study indicates the importance of DNA binding residues within loop 2 for NFκBp50-*MYB* SLR polyU complex formation. Our mutational analysis suggests that at least in the case of Arg 56, loop 1 residues do not make a significant contribution to the NFκBp50-*MYB* SLR polyU complex.

It is important to note that while our data are consistent with the idea that the *MYB* SLR polyU acts as a scaffold to recruit RNA binding factors such as NFκBp50, it is likely that these proteins function with additional co-factors in order to promote elongation through intron 1. P-TEFb (CDK9/ CyclinT1) is an essential co-factor for the Tat-*TAR* RNA complex with the CDK9 component playing a key role in mediating the effects of Tat on transcriptional elongation [57–59]. Given our observations that NFκBp50-driven *MYB* elongation is CDK9 dependent, it is possible that a similar mechanism may exist for the *MYB* SLR polyU involving an NFκBp50-SLR-P-TEFb complex. Our data also show a role for NFκBp65 (RelA) in regulating transcriptional elongation through intron 1 of *MYB* in a SLR and polyU tract dependent manner. RNA shift analysis indicated that NFκBp65 did not bind efficiently to *MYB* SLR polyU RNA. These observations suggest that NFκBp65 might be recruited to *MYB* SLR polyU via its association with NFκBp50 that is bound to the SLR. Indeed this scenario is reminiscent of a large body of published data that have shown that NFκBp65 binds DNA poorly but elicits its transcriptional activity by forming a heterodimer with NFκBp50 on promoter DNA [32, 36]. While we were unable to detect NFκBp50-65 heterodimers in our RNA experiments, our gene reporter assays suggest that NFκBp50-65 fusion protein in which the NFκBp65 AD is tethered to the NFκBp50 RHD, is a potent activator of *MYB* elongation via the *MYB* SLR and polyU tract. Collectively these data support the idea that NFκBp65 can activate the *MYB* SLR polyU without directly binding the *MYB* SLR polyU. Interestingly, NFκBp65 has been shown to mediate transcriptional elongation through the direct recruitment of P-TEFb [50]. Taken together, these observations invoke a model (Fig 8) where the NFκBp50-p65-SLR complex contributes to the stimulation of *MYB* transcription elongation by the NFκBp65-mediated recruitment of P-TEFb and the subsequent Ser2 phosphorylation of PolII CTD (elongating form) by CDK9. This model is consistent with our recent observations that ERα recruits P-TEFb to a region near the *MYB* SLR polyU and that this interaction is concordant with the accumulation of Ser2 CTD phosphorylated pol II bound to this region and the relief of *MYB* transcriptional attenuation [22]. In addition, the conservation of key amino acid residues required for *MYB* SLR polyU binding among NFκB family members suggests that alternative combinations of NFκB complexes may form with the *MYB* SLR polyU. This hypothesis raises the intriguing possibility that the formation of multiple types of NFκB-*MYB* SLR polyU complexes may elicit specific effects on *MYB* elongation and expression. Future studies identifying other NFκB components—and indeed other factors—associated with *MYB* SLR polyU and confirming the role of P-TEFb may shed light on the function of these complexes in cellular processes where control of *MYB* expression is critical.

## Supporting Information

S1 Fig Western blot analysis confirms ectopic protein expression from mammalian expression vectors. Protein extracts were resolved on NuPAGE 4–12% PAGE gels in MOPs buffer and transferred to PVDF membrane. (A) Related to Fig 7. Western blot analysis of nuclear extracts confirms the expression of Tat101 in transfected 293 cells. Tat expression was not detected in untransfected cells. Tat101 protein was detected using anti-HIV-1 Tat (ab42359). (B) Related to Fig 7. Western blot analysis of nuclear extracts confirms the expression of pCMV driven CDK9 and DNCDK9 in transfected 293 cells. CDK9 expression was not detected in untransfected cells. CDK9 proteins were detected using anti-CDK9 (L19; SC). (C) Related to Fig 4. Western blot analysis of nuclear extracts confirms the expression of V5 tagged NFκBp50 and NFκBp65 in transfected 293 cells. NFκBp50 and NFκBp65 expression was not detected in untransfected cells. V5 tagged NFκBp50 and NFκBp65 proteins were detected using anti-V5 (V5-10; Sigma). (D) Related to Figs 4 and 5. Western blot analysis of nuclear extracts confirms the expression of the NFκBp50-p65 fusion and its mutated derivatives in transfected 293 cells. NFκBp50-p65 expression was not detected in untransfected cells. NFκBp50-p65 proteins were detected using anti-NFκBp50 (E10; SC) and anti-NFκBp65 (C20; SC). In order to account for differences in the levels of expression between NFκBp50-p65 and its mutated forms, plasmid amounts were adjusted where indicated in the Figure legends.
(TIF)

S2 Fig *MYB* SLR polyU structure analyses. (A) Schematic outlining the position of the 3L and 15C1 mutations within the *MYB* SLR polyU [21]. The predicted secondary structure of the *MYB* SLR mutants by MFold analysis is shown. (B) Electrophoretic mobility of radiolabeled *MYB* SLR RNA versus *MYB* SLR 3L mutation and *MYB* SLR 15C1 mutation RNA transcripts generated from *MYB* SLR polyU templates (pBluescript II KS). RNAs were subjected to electrophoresis in a denaturing (6M Urea), 4% acrylamide gel where probes migrate according to size and a 4% non-denaturing acrylamide gel where secondary structure is maintained.
(TIF)

S3 Fig NFκBp50 Rel homology domain (RHD) DNA binding and transactivation activity. (A) EMSA IgκB DNA probe [32] was incubated with purified NFκB p50 RHD (aa 42–365) or corresponding mutants and the p50 RHD-DNA complexes resolved on a 6% non-denaturing polyacrylamide/bisacrylamide (29:1) gel buffered with 0.5 X TBE. The black arrows indicate the position of the NFκB RHD-DNA complex; the grey arrow indicates free IgκB DNA probe. (B) NFκBp50 R56A-p65 does not induce endogenous *MYB*. Total RNA was isolated from 293 cells transfected with; 1 μg of pcDNA NFκB p50-p65 or 1 μg of pcDNA NFκBp50 R56A and analyzed by Q-PCR to measure *MYB* expression levels. Error bars represent mean ± SEM of n = 2 experiments. (C) Transactivation studies in 293 cells using 2 μg of the *MYB* SLR polyU CAT and 0.25 μg of pcDNA NFκBp50-p65 or 0.25 μg of pcDNA NFκBp50 (R56A)-p65. Error bars represent mean ± SEM, * P <0.05.
(TIF)

S4 Fig NFκBp50 and p65 binding to *MYB* SLR polyU RNA. (A) Right panel. Western blot analysis of the recombinant NFκBp65 preparation (Origene). NFκBp65 (25 ng, 50 ng and 100 ng) was resolved on a NuPAGE 4–12% PAGE in MOPs buffer and transferred to PVDF membrane. Membranes were probed with anti-NFκBp65 (A) or anti-NFκBp65 (C20) and developed with HRP secondary antibodies. Left panel. An RNA probe generated from *pGEM-3Zf MYB* SLR polyU was incubated with 12.5, 25, 50 and 100 ng of recombinant NFκBp50 (Panomics) or NFκBp65 (Origene) and the reactions resolved on a 5% Tris-glycine gel. The black arrow indicates the position of the NFκBp50 or p65-*MYB* SLR polyU RNA complexes; the grey arrow indicates free probe. (B) NFκBp65 DNA binding activity. Left panel. EMSA HIV-1 LTR DNA probe [33] was incubated with 100 ng of purified NFκBp65 and the NFκBp65-DNA complexes resolved on a 6% non-denaturing polyacrylamide/bisacrylamide (29:1) gel buffered with 0.5 X TBE. NFκBp65-DNA complexes were super-shifted by the addition of anti-NFκBp65 antibody. Anti-NFκBp50 and anti-Myc IgG was used as a control. The black arrows indicate the position of the NFκBp65-DNA complex; the grey arrow indicates free HIV-1 LTR DNA probe. The white arrows show the position of the complexes in the presence of the antibody. Right panel. EMSA HIV-1 LTR DNA probe [33] was incubated with 100 ng of purified NFκBp65 with or without the addition of 10 ng purified NFκBp50 and the NFκB-DNA complexes resolved on a 6% non-denaturing polyacrylamide/bisacrylamide (29:1) gel buffered with 0.5 X TBE. NFκB-DNA complexes were super-shifted by the addition of anti-NFκBp65 or anti-NFκBp50 antibody. Anti-Myc IgG was used as a control. The black arrows indicate the position of the NFκB-DNA complexes; the grey arrow indicates free HIV-1 LTR DNA probe. The white arrows show the position of the complexes in the presence of the antibody. (C) Left panel. NFκBp65 (Origene) was titrated into NFκBp50-*MYB* SLR polyU binding reactions and the reactions resolved on a 5% Tris-glycine gel. Reactions contained 12.5 ng of recombinant NFκBp50 (Panomics) and 0, 12.5, 25, 50 or 100 ng of recombinant NFκBp65 (Origene). The black arrow indicates the position of the NFκBp50-*MYB* SLR polyU RNA complex. The grey arrow indicates free probe. Right Panel. Quantitation of NFκBp50-*MYB* SLR polyU complexes across triplicate experiments by PhosphorImage analysis. NS; not statistically significant.
(TIF)

S5 Fig Binding of NFκBp50 to HIV *TAR* RNA. (A) Left. HIV *TAR* RNA probe was incubated with 50 ng of recombinant NFκBp50 and reactions resolved on a 5% Tris-glycine gel. The black arrow indicates the position of the NFκB p50-*TAR* RNA complex; the grey arrow indicates free *TAR* RNA probe. Right. Transactivation studies in 293 cells using 2 μg of *MYB* TAR CAT and 0.25 μg of pcDNA NFκBp50-p65. (B) Long range interaction between the *MYB* promoter and intron 1 *MYB* SLR polyU region. 3C analysis of the *MYB* promoter and intron 1 regions *in vivo* was performed using Colo201 cells in the presence or absence of sodium butyrate (20 μM; 6 h). A schematic of the *MYB* gene is shown with the BamH1 sites that were exploited in the 3C assay. Primers were located in the 5´ regions flanking the BamH1 sites to allow identification of long range interaction between the *MYB* promoter and intron 1 *MYB* SLR polyU region. Error bars represent mean ± SEM, * P <0.05.
(TIF)

S1 Table Oligonucleotide sequences used in this study. Oligonucleotide sequences were purchased from Geneworks (Australia) and used as primers in quantitative RT-PCRs or as probes in electrophoretic mobility shift assays.
(DOCX)
