# Supplementary figures and images for: MYB Elongation Is Regulated by the Nucleic Acid Binding of NFκB p50 to the Intronic Stem-Loop Region
Source: PLoS One. 2015 Apr 8;10(4):e0122919. doi: 10.1371/journal.pone.0122919 (PMC4390348; doi:10.1371/journal.pone.0122919)

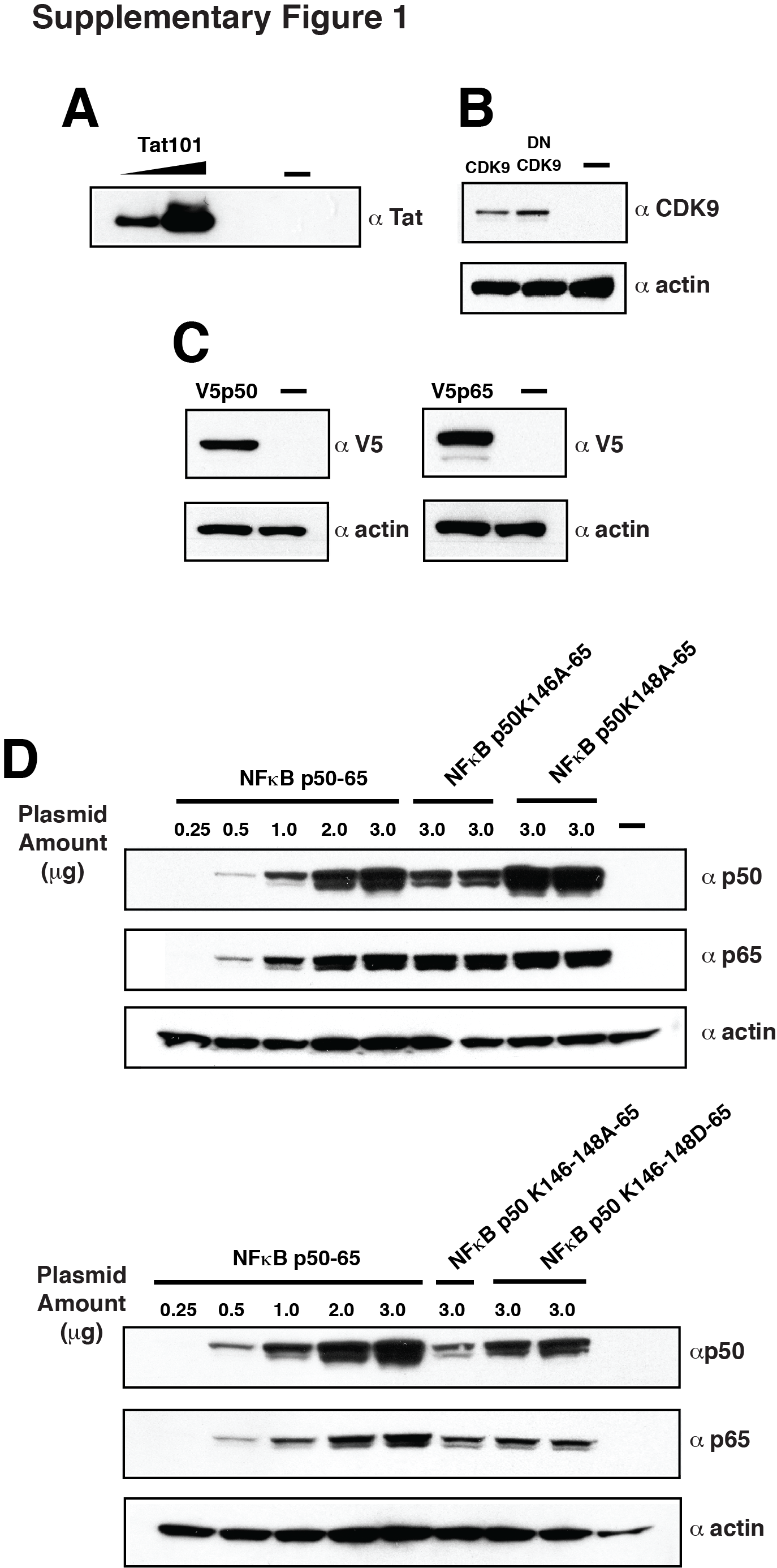

Supplement: S1 Fig — Protein extracts were resolved on NuPAGE 4–12% PAGE gels in MOPs buffer and transferred to PVDF membrane. (A) Related to Fig 7. Western blot analysis of nuclear extracts confirms the expression of Tat101 in transfected 293 cells. Tat expression was not detected in untransfected cells. Tat101 protein was detected using anti-HIV-1 Tat (ab42359). (B) Related to Fig 7. Western blot analysis of nuclear extracts confirms the expression of pCMV driven CDK9 and DNCDK9 in transfected 293 cells. CDK9 expression was not detected in untransfected cells. CDK9 proteins were detected using anti-CDK9 (L19; SC). (C) Related to Fig 4. Western blot analysis of nuclear extracts confirms the expression of V5 tagged NFκBp50 and NFκBp65 in transfected 293 cells. NFκBp50 and NFκBp65 expression was not detected in untransfected cells. V5 tagged NFκBp50 and NFκBp65 proteins were detected using anti-V5 (V5-10; Sigma). (D) Related to Figs 4 and 5. Western blot analysis of nuclear extracts confirms the expression of the NFκBp50-p65 fusion and its mutated derivatives in transfected 293 cells. NFκBp50-p65 expression was not detected in untransfected cells. NFκBp50-p65 proteins were detected using anti-NFκBp50 (E10; SC) and anti-NFκBp65 (C20; SC). In order to account for differences in the levels of expression between NFκBp50-p65 and its mutated forms, plasmid amounts were adjusted where indicated in the Figure legends. (TIF) [file pone.0122919.s001.tif]

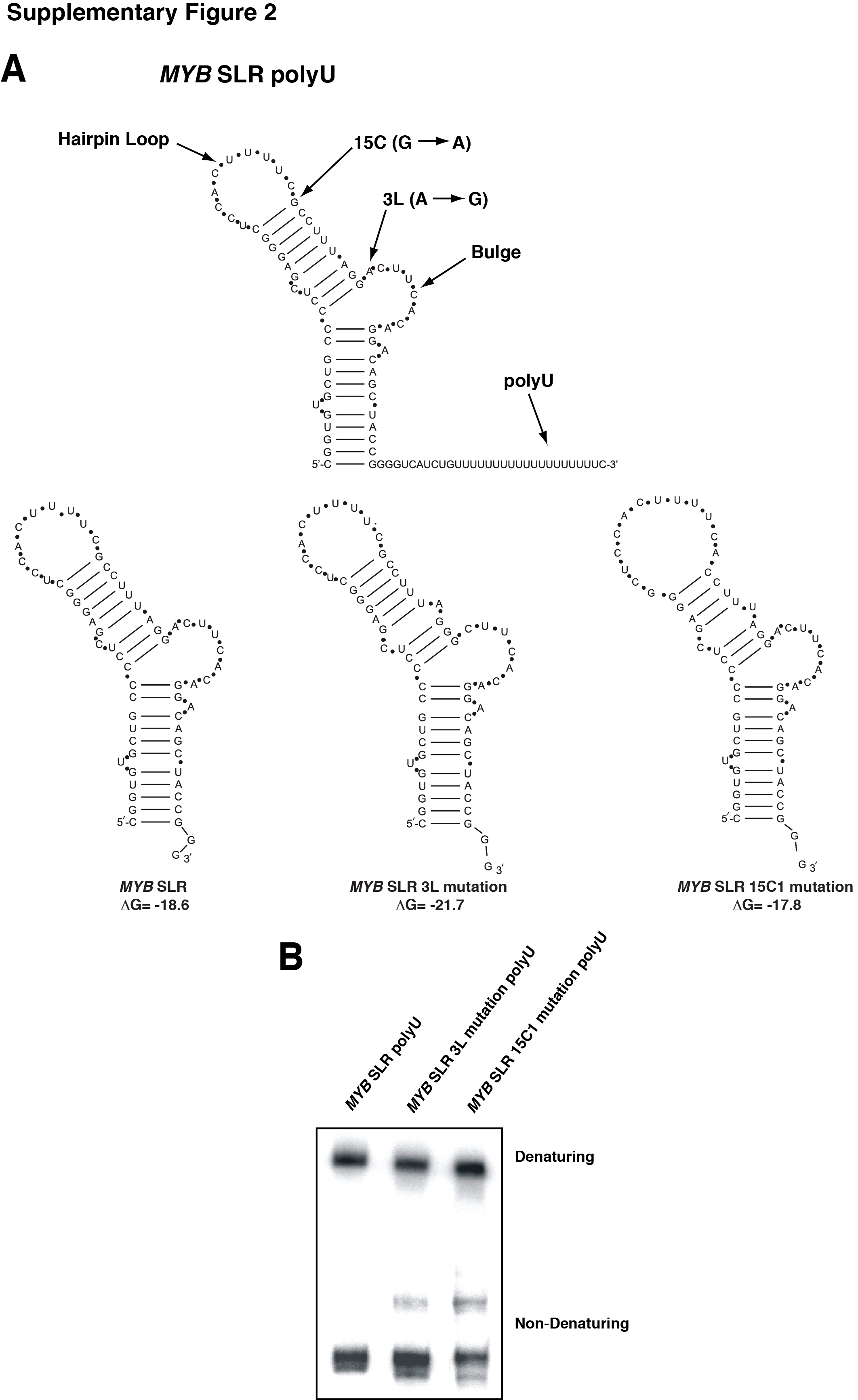

Supplement: S2 Fig — (A) Schematic outlining the position of the 3L and 15C1 mutations within the MYB SLR polyU [21]. The predicted secondary structure of the MYB SLR mutants by MFold analysis is shown. (B) Electrophoretic mobility of radiolabeled MYB SLR RNA versus MYB SLR 3L mutation and MYB SLR 15C1 mutation RNA transcripts generated from MYB SLR polyU templates (pBluescript II KS). RNAs were subjected to electrophoresis in a denaturing (6M Urea), 4% acrylamide gel where probes migrate according to size and a 4% non-denaturing acrylamide gel where secondary structure is maintained. (TIF) [file pone.0122919.s002.tif]

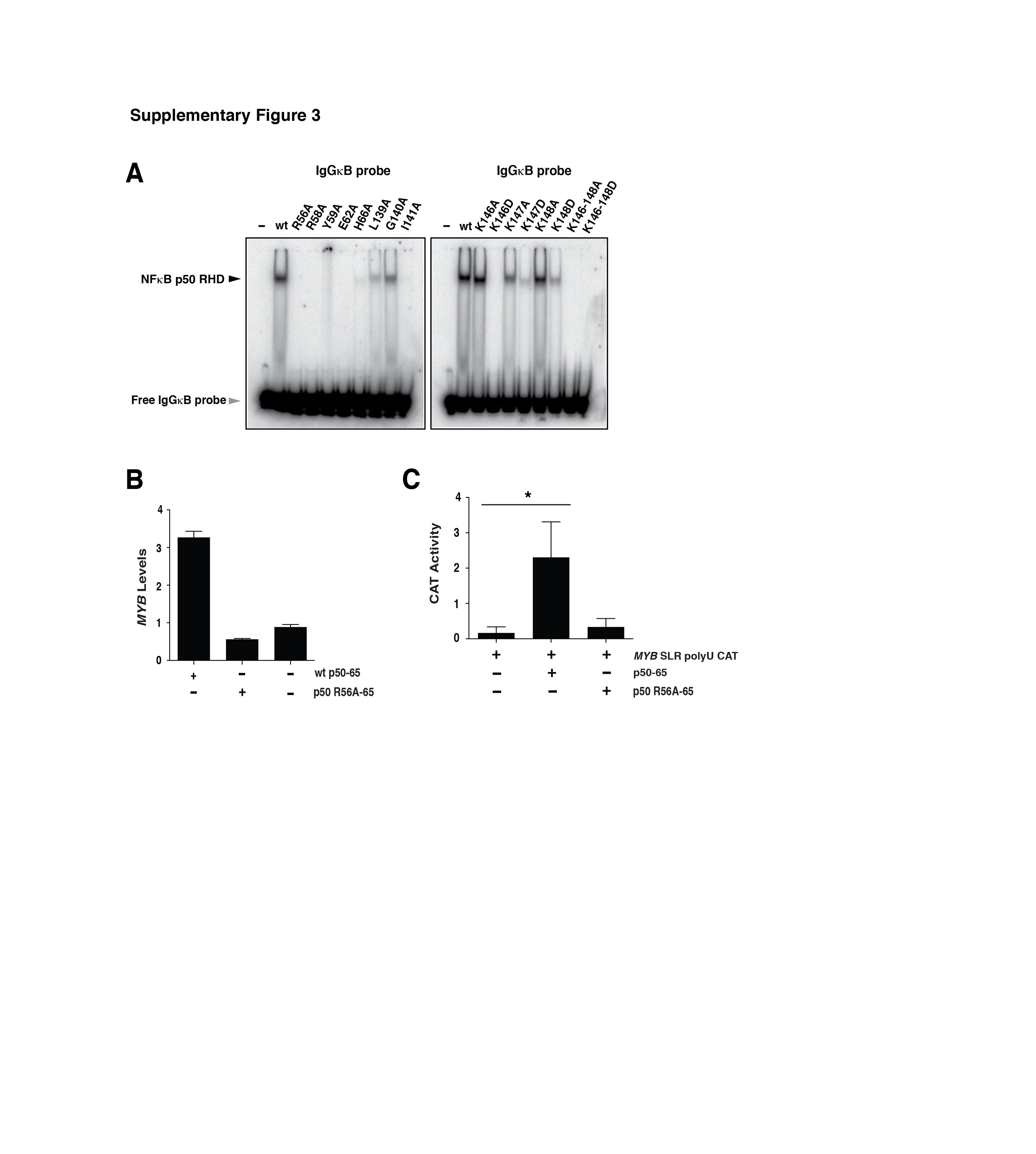

Supplement: S3 Fig — (A) EMSA IgκB DNA probe [32] was incubated with purified NFκB p50 RHD (aa 42–365) or corresponding mutants and the p50 RHD-DNA complexes resolved on a 6% non-denaturing polyacrylamide/bisacrylamide (29:1) gel buffered with 0.5 X TBE. The black arrows indicate the position of the NFκB RHD-DNA complex; the grey arrow indicates free IgκB DNA probe. (B) NFκBp50 R56A-p65 does not induce endogenous MYB. Total RNA was isolated from 293 cells transfected with; 1 μg of pcDNA NFκB p50-p65 or 1 μg of pcDNA NFκBp50 R56A and analyzed by Q-PCR to measure MYB expression levels. Error bars represent mean ± SEM of n = 2 experiments. (C) Transactivation studies in 293 cells using 2 μg of the MYB SLR polyU CAT and 0.25 μg of pcDNA NFκBp50-p65 or 0.25 μg of pcDNA NFκBp50 (R56A)-p65. Error bars represent mean ± SEM, * P <0.05. (TIF) [file pone.0122919.s003.tif]

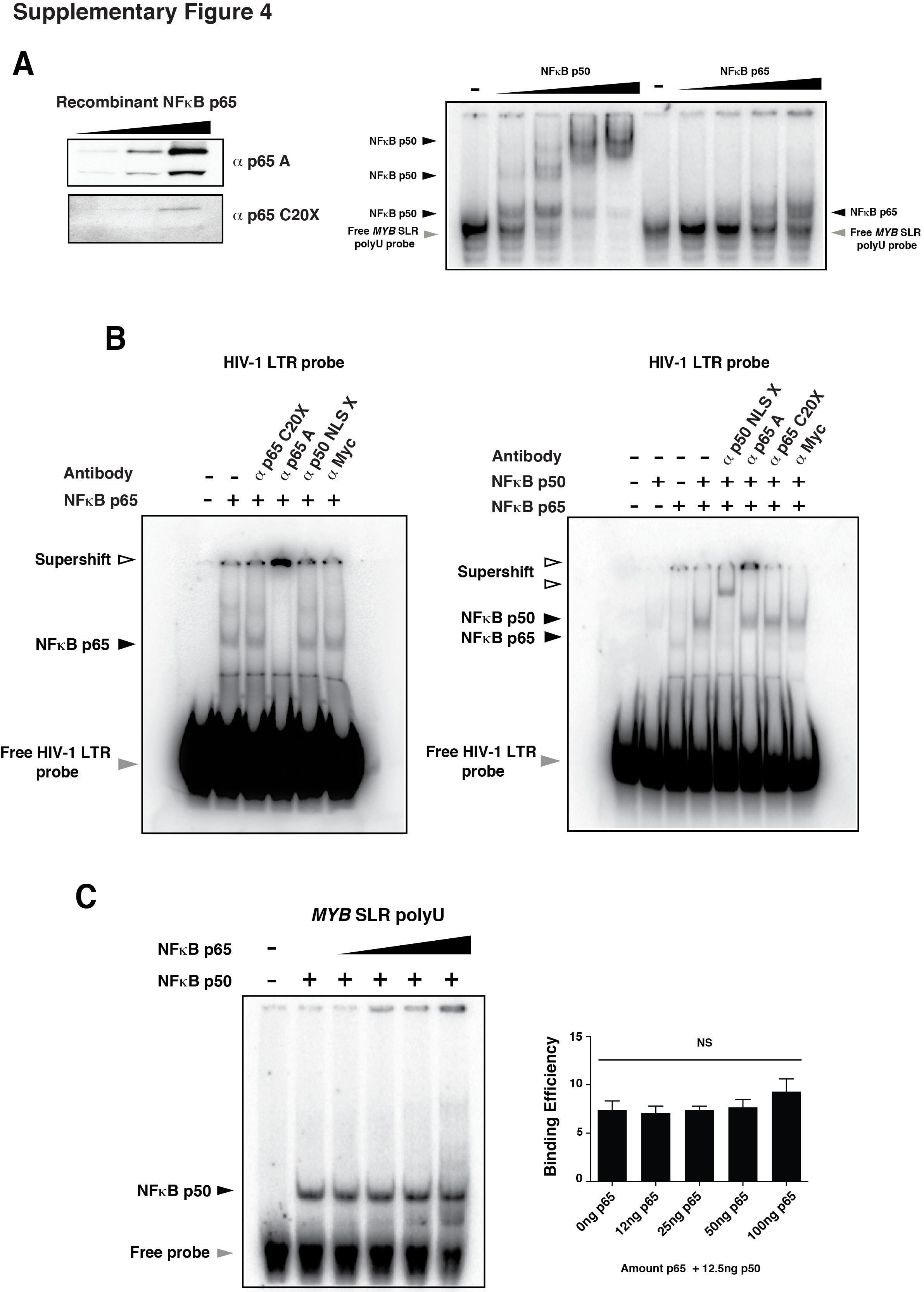

Supplement: S4 Fig — (A) Right panel. Western blot analysis of the recombinant NFκBp65 preparation (Origene). NFκBp65 (25 ng, 50 ng and 100 ng) was resolved on a NuPAGE 4–12% PAGE in MOPs buffer and transferred to PVDF membrane. Membranes were probed with anti-NFκBp65 (A) or anti-NFκBp65 (C20) and developed with HRP secondary antibodies. Left panel. An RNA probe generated from pGEM-3Zf MYB SLR polyU was incubated with 12.5, 25, 50 and 100 ng of recombinant NFκBp50 (Panomics) or NFκBp65 (Origene) and the reactions resolved on a 5% Tris-glycine gel. The black arrow indicates the position of the NFκBp50 or p65-MYB SLR polyU RNA complexes; the grey arrow indicates free probe. (B) NFκBp65 DNA binding activity. Left panel. EMSA HIV-1 LTR DNA probe [33] was incubated with 100 ng of purified NFκBp65 and the NFκBp65-DNA complexes resolved on a 6% non-denaturing polyacrylamide/bisacrylamide (29:1) gel buffered with 0.5 X TBE. NFκBp65-DNA complexes were super-shifted by the addition of anti-NFκBp65 antibody. Anti-NFκBp50 and anti-Myc IgG was used as a control. The black arrows indicate the position of the NFκBp65-DNA complex; the grey arrow indicates free HIV-1 LTR DNA probe. The white arrows show the position of the complexes in the presence of the antibody. Right panel. EMSA HIV-1 LTR DNA probe [33] was incubated with 100 ng of purified NFκBp65 with or without the addition of 10 ng purified NFκBp50 and the NFκB-DNA complexes resolved on a 6% non-denaturing polyacrylamide/bisacrylamide (29:1) gel buffered with 0.5 X TBE. NFκB-DNA complexes were super-shifted by the addition of anti-NFκBp65 or anti-NFκBp50 antibody. Anti-Myc IgG was used as a control. The black arrows indicate the position of the NFκB-DNA complexes; the grey arrow indicates free HIV-1 LTR DNA probe. The white arrows show the position of the complexes in the presence of the antibody. (C) Left panel. NFκBp65 (Origene) was titrated into NFκBp50-MYB SLR polyU binding reactions and the reactions resolved on a 5% Tris-glycine gel. React [file pone.0122919.s004.tif]

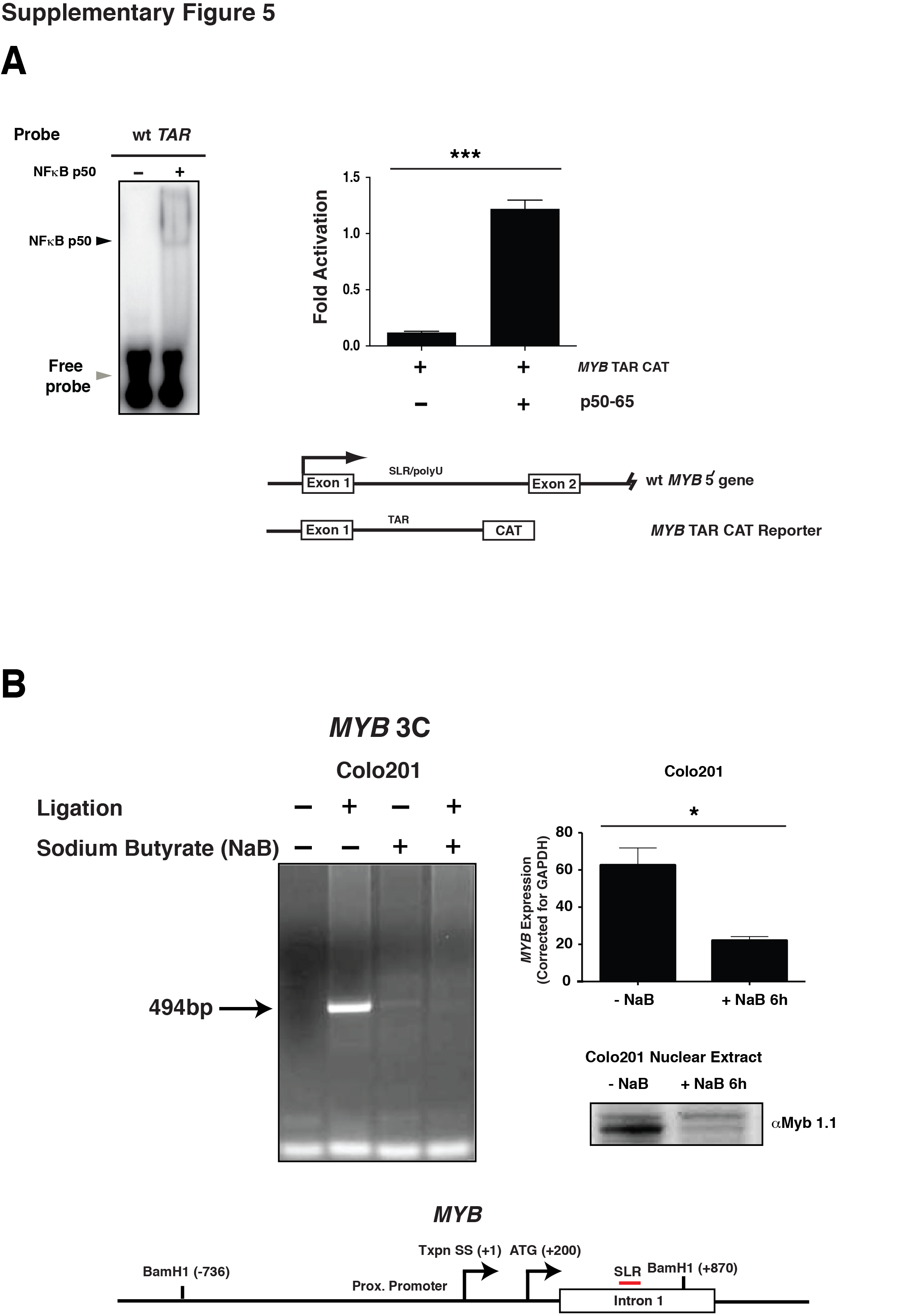

Supplement: S5 Fig — (A) Left. HIV TAR RNA probe was incubated with 50 ng of recombinant NFκBp50 and reactions resolved on a 5% Tris-glycine gel. The black arrow indicates the position of the NFκB p50-TAR RNA complex; the grey arrow indicates free TAR RNA probe. Right. Transactivation studies in 293 cells using 2 μg of MYB TAR CAT and 0.25 μg of pcDNA NFκBp50-p65. (B) Long range interaction between the MYB promoter and intron 1 MYB SLR polyU region. 3C analysis of the MYB promoter and intron 1 regions in vivo was performed using Colo201 cells in the presence or absence of sodium butyrate (20 μM; 6 h). A schematic of the MYB gene is shown with the BamH1 sites that were exploited in the 3C assay. Primers were located in the 5´ regions flanking the BamH1 sites to allow identification of long range interaction between the MYB promoter and intron 1 MYB SLR polyU region. Error bars represent mean ± SEM, * P <0.05. (TIF) [file pone.0122919.s005.tif]
