# Supplementary material for: MYB Elongation Is Regulated by the Nucleic Acid Binding of NFκB p50 to the Intronic Stem-Loop Region
Source: PLoS One. 2015 Apr 8;10(4):e0122919. doi: 10.1371/journal.pone.0122919 (PMC4390348; doi:10.1371/journal.pone.0122919)
Supplement: S1 Table — Oligonucleotide sequences were purchased from Geneworks (Australia) and used as primers in quantitative RT-PCRs or as probes in electrophoretic mobility shift assays. (DOCX) [file pone.0122919.s006.docx]

**Supplementary Table 1.** Oligonucleotide sequences used in this study. Oligonucleotide sequences were purchased from Geneworks (Australia) and used as primers in quantitative RT-PCRs or as probes in electrophoretic mobility shift assays.

| **Primer** | **Sequence** |
| --- | --- |
| ***RT-PCR-GAPDH transcripts*** |  |
| 5′ primer | ACCACCATGGAGAAGGC |
| 3′ primer | CTCAGTGTAGCCCAGGATGC |
| ***RT-PCR-MYB transcripts*** |  |
| 5′ primer | GCTTCCCAAGTCTGGAAAGCG |
| 3′ primer | GAAGACTCCTGCAGATAACC |
| ***RT-PCR-MYB***  ***Pre/PostSLR transcripts***  ***(Fig. 6D)*** |  |
| 5′ Pre SLR Primer | GGGAAATCCTCGTCCGAACTGTCAG |
| 3′ Pre SLR Primer | TGAAGGAGGGTGCGAGAA |
| 5′ Post SLR Primer | TTAACCAGGTCAGCGAAATG |
| 3′ Post SLR Primer | TGCGATTTGCTTGCTCTAAGT |
| ***Q-PCR-MYB***  ***Pre/PostSLR transcripts***  ***(Fig. 5E, G-I)*** |  |
| 5′ Pre SLR Primer | GAAATCCTCGTCCGAACTGTCAG |
| 3′ Pre SLR Primer | GCGTGTGCTGCTGGGAAAG |
| 5′ Post SLR Primer | CCTCCGAATCACAGTAGC |
| 3′ Post SLR Primer | TTCTGTCAAGGAAACAAACC |
| ***DNA EMSA-probes*** |  |
| IgκB PS | TCTGAGGGACTTTCCT |
| IgκB AS  ***NFκB p50 mutagenesis*** | GATCAGGAAAGTCCCT |
| p50 R54A PS | AACCTAAACAGAGAGGATTTGCTTTCCGTTATG |
| p50 R54A AS | AAATCCTCTCTGTTTAGGTTGCTCTAATAT |
| p50 R56A PS | AACAGAGAGGATTTCGTTTCGCTTATGTATGTG |
| p50 R56A AS | GAAACGAAATCCTCTCTGTTTAGGTTGCTC |
| p50 E60A PS | TTCGTTTCCGTTATGTATGTGCAGGCCCATCCC |
| p50 E60A AS | CACATACATAACGGAAACGAAATCCTCTCTG |
| p50 H64A PS | ATGTATGTGAAGGCCCATCCGCTGGTGGACTAC |
| p50 H64A AS | GGATGGGCCTTCACATACATAACGGAAACG |
| p50 L137A PS | TGGTGGTCGGCTTCGCAAACGCGGGTATACTTC |
| p50 L137A AS | GTTTGCGAAGCCGACCACCATGTCCTTGGG |
| p50 G138A PS | TGGTCGGCTTCGCAAACCTGGCTATACTTCATG |
| p50 G138A AS | CCAGGTTTGCGAAGCCGACCACCATGTCCTT |
| p50 I139A PS | TCGGCTTCGCAAACCTGGGTGCACTTCATGTGA |
| p50 I139A AS | ACCCAGGTTTGCGAAGCCGACCACCATGTC |
| p50 K144A PS | TGGGTATACTTCATGTGACAGCGAAAAAAGTAT |
| p50 K144D PS | TGGGTATACTTCATGTGACAGATAAAAAAGTAT |
| p50 K144A AS | TGTCACATGAAGTATACCCAGGTTTGCGAA |
| p50 K145A PS | GTATACTTCATGTGACAAAGGCAAAAGTATTTG |
| p50 K145D PS | GTATACTTCATGTGACAAAGGATAAAGTATTTG |
| p50 K145A AS | CTTTGTCACATGAAGTATACCCAGGTTTG |
| p50 K146A PS | ACTTCATGTGACAAAGAAAGCAGTATTTGAAA |
| p50 K146D PS | TACTTCATGTGACAAAGAAAGATGTATTTGAAA |
| p50 K146A AS | TTTCTTTGTCACATGAAGTATACCCAGGTT |
| p50 K145146A PS | GTATACTTCATGTGACAGCGGCAGCAGTATTTGAAACAC |
| p50 K145146A AS | CGCTGTCACATGAAGTATACCCAGGTTTGCGAA |
| p50 K145146D PS | GTATACTTCATGTGACAGATGATGATGTATTTGAAACAC |
| p50 K145146D AS | ATCTGTCACATGAAGTATACCCAGGTTTGCGAA |
| ***RNA ChIP analysis*** |  |
| 5′ Pre SLR Primer | CTCTGGGGACGAGAGGGCGACTT |
| 3′ SLR Primer | GCAGCACACCGTCCTGCG |
